# Reciprocal modulation between beef protein and wine phenolics during *in vitro* co-digestion: a proposed ternary complex-mediated mechanism modulated by cooking technique

**DOI:** 10.3389/fnut.2026.1967708

**Published:** 2026-10-14

**Authors:** Ziruo Sun, Shaoyun Xing, Weixiang Tang, Taihong Liu, Kaiyue Liu, Chao Du, Shuyang Sun, Wenguang Jiang

**Affiliations:** 1 School of Food Engineering, Ludong University, Yantai, Shandong, China; 2 Ningxia Changyu Longyu Estate Co. Ltd., Yinchuan, Ningxia, China; 3 College of Enology, Northwest A&F University, Yangling, Shaanxi, China

**Keywords:** beef–wine co-digestion, cooking technique, phenolic bioaccessibility, protein digestibility, protein-phenolics interaction

## Abstract

**Background:**

Beef and red wine form a classic dietary combination, yet how their components reciprocally influence each other during digestion remains unclear.

**Methods:**

This study co-digested steamed, boiled and grilled beef with red wine using the INFOGEST 2.0 *in vitro* digestion model, combined with molecular docking, to investigate bidirectional modulation between beef proteins and wine phenolics.

**Results:**

Wine co-digestion suppressed gastric carbonyl formation by 21.5%−30.8% and preserved free sulfhydryl groups by 27.7%−50.5% across cooking methods. Two-way analysis of variance indicated significant cooking × wine interactions for protein oxidation and amino acid release. The largest improvement occurred in grilled beef, whose gastric digestibility and intestinal amino acid content increased by 20.9% and 67.2%, respectively. Docking simulations predicted that selected phenolics bound to myosin heavy chain and pepsin through hydrogen bonding and hydrophobic interactions, supporting a proposed phenolics-pepsin-myosin ternary complex that may strengthen enzyme-substrate association. Reciprocally, the beef matrix enhanced ABTS antioxidant activity by 24.9% (gastric) and 125.3% (intestinal) relative to wine alone, and was associated with selective phenolic regulation: gastric gallic acid increased by up to 135.7%, whereas intestinal epicatechin and resveratrol rose up to 3.5- and 11.6-fold, respectively.

**Conclusion:**

These findings support the proposed ternary complex as a plausible molecular hub governing protein–phenolic reciprocity, providing a proposed mechanistic basis for optimizing cooking methods and meat–wine pairing strategies in mixed meals.

## 1 Introduction

Beef is a major dietary source of high-quality protein, yet its nutritional value is significantly affected by cooking processes (1, 2). Thermal processing induces protein oxidation and structural unfolding, enhances microbiological safety, and improves sensory attributes such as flavor and texture (3). A few studies have shown that cooking methods such as roasting and boiling differentially affect meat quality, due to variations in heat transfer efficiency, temperature distribution, and the chemical environment during processing (4, 5). These method-dependent alterations in protein physicochemical properties will in turn influence subsequent proteolytic digestion and the bioavailability of amino acids (6, 7).

Wine, commonly consumed alongside beef, contains a wide range of phenolics associated with health benefits. These compounds exhibit potent antioxidant activities, including radical scavenging, inhibition of lipid peroxidation, and metal chelation (8). In simulated digestion systems, red wine has been shown to alleviate oxidative stress and to exhibit greater antioxidant capacity than white wine under digestive conditions, with the capacity strongly correlated with total phenolics, flavonoids, flavanols and anthocyanins (9, 10).

Despite these advances, critical knowledge gaps remain. Existing studies have largely investigated beef digestion in isolation or focused on the singular effects of cooking methods, while wine phenolics have been examined primarily as independent dietary components. Few studies have considered the complex interactions that occur when cooked beef and wine are co-digested as a mixed meal, which is a scenario that closely mirrors real-world dietary patterns. To address these knowledge gaps, the present study investigates the effects of three cooking methods (steaming, boiling, and grilling) on the protein oxidation and digestibility of beef, as well as the modulatory role of red wine phenolics within the context of a mixed meal, during *in vitro* gastrointestinal digestion. The main objectives are as follows: (1) to elucidate the impact of wine phenolics on beef protein oxidation, digestibility and amino acid release during co-digestion; (2) to compare the effects of different cooking methods on the aforementioned protein parameters; and (3) to evaluate the influence of the beef protein matrix on the content and bioaccessibility of wine derived phenolic compounds. By proposing an integrated framework that correlates cooking-induced oxidative status, mixed-meal co-digestion, and reciprocal protein–phenolic interactions, this study aims to provide a mechanistic framework for understanding the potential bidirectional modulation within the gastrointestinal tract. The findings are expected to guide the rational design of mixed meals containing cooked meat and alcoholic beverage, thereby improving protein bioavailability and enhancing antioxidant protection during digestion.

## 2 Materials and methods

### 2.1 Materials and reagents

A commercially available red wine (Yellow Tail, 2020 vintage, Australia) with an ethanol content of 13.0% (v/v) was used. Enzymes required for the *in vitro* simulated digestion including α-amylase, pepsin, gastric lipase, pancreatin, and bile salts, were purchased from Sigma-Aldrich (St. Louis, MO, USA). All other chemical reagents were of analytical grade and sourced from Sinopharm Chemical Reagent Co., Ltd. (Shanghai, China).

### 2.2 Beef preparation

Beef (*Longissimus dorsi* muscle) was obtained from a local market. Following slaughter, the carcass was aged at 4 °C for 72 h. The muscle was then portioned, vacuum-packed, and stored at −20 °C until required. Prior to cooking, samples were thawed at 4 °C overnight. Beef was sliced into uniform 1 cm thick portions before thermal processing. Three cooking treatments were applied: steaming (ST), in which samples were steamed over boiling water (100 °C) for 10 min; boiling (BT), in which samples were fully submerged in boiling water (100 °C) for 10 min; and grilling (GT), in which samples were cooked on a preheated griddle for 90 s per side to achieve a well-done level of doneness. The cooking endpoint was standardized by monitoring the core temperature at the geometric center of each portion; all samples had reached 71 °C~74 °C at the end of cooking. All samples were cooled to ambient temperature. Surface moisture was removed using absorbent paper, and 5 g aliquots were precisely weighed for subsequent *in vitro* digestion experiments.

### 2.3 Experimental sample design and *in vitro* digestion simulation

The experimental design included mixed-meal samples and two kinds of controls. Mixed meal: each sample contained 5 g of cooked beef (prepared from ST, BT or GT) combined with 10 ml of red wine, designated as STW, BTW and GTW, respectively. Control I consisted of 5 g cooked beef with 10 mL of deionized water (designated as STC, BTC and GTC, respectively). Control II consisted of 10 mL wine alone. The beef/wine ratio (1:2, w/v) was selected to maintain sufficient fluidity and homogeneous mixing of the digesta during the static *in vitro* gastrointestinal simulation, thereby ensuring reproducible enzyme-substrate contact under the INFOGEST 2.0 protocol. This ratio also ensured that wine-derived phenolics in the post-digestion supernatants remained detectable and quantifiable under the HPLC-UV and antioxidant assay conditions. Although the wine fraction is higher than that of a typical dietary serving, the mixture was designed as a wine-rich model meal to compensate for the small absolute beef mass and to enable mechanistic comparison, rather than to reproduce a standard serving exactly.

A static *in vitro* digestion simulation was performed according to the standardized INFOGEST 2.0 protocol (11) with slight modifications. Briefly, each sample was mixed with 15 ml of simulated salivary fluid, and the pH was adjusted to 7.0. α-Amylase was added to achieve a final activity of 75 U/ml. Oral digestion was simulated at 37 °C for 2 min under constant agitation. The resulting oral solution was then mixed with an equal volume of simulated gastric fluid. The pH was adjusted to 3.0, and pepsin (2,000 U/ml) along with gastric lipase (60 U/ml) were introduced. Gastric digestion was conducted at 37 °C for 2 h. Subsequently, the pH of the gastric chyme was raised to 6.0 and mixed 1:1 (v/v) with simulated intestinal fluid. The pH was further adjusted to 7.0. Pancreatin (100 U/ml, calculated as trypsin activity) and bile salts (10 mM final concentration) were added to initiate the intestinal phase, which proceeded at 37 °C for 2 h. Digesta from both the gastric and intestinal phases were collected separately, and immediately centrifuged (10,000 × g, 20 min, 4 °C). The resulting supernatants were lyophilized and stored at −80 °C for subsequent analyses. A digestion blank (containing the full digestive fluids and enzymes but no beef or wine) was run in parallel, and its background values were subtracted from the protein, carbonyl and sulfhydryl measurements.

### 2.4 Protein oxidation analysis

#### 2.4.1 Quantification of protein carbonyl content

Protein carbonyl content was determined according to the DNPH derivatization method described by Han et al. (12) with slight modifications. In brief, digested samples were homogenized in a chilled sodium pyrophosphate buffer (50 mM, pH 6.5). Proteins were subsequently precipitated using 40% (w/v) trichloroacetic acid (TCA). The resulting pellet was incubated with 10 mM DNPH dissolved in 2 M HCl for 60 min at 30 °C under dark conditions. To eliminate any unreacted DNPH, the pellet was washed multiple times with a 1:1 (v/v) mixture of ethanol and ethyl acetate. The derivatized proteins were then solubilized in 6 M guanidine hydrochloride (pH 2.3). Absorbance readings were taken at 370 nm. The protein concentration in the final solution was quantified via the bicinchoninic acid (BCA) assay. The carbonyl content was determined using a molar extinction coefficient of 22,000 L·mol^−1^·cm^−1^ and reported as micromoles of carbonyl per gram of lyophilized digesta (μmol/g).

#### 2.4.2 Determination of free sulfhydryl (SH) group content

The free sulfhydryl (SH) content was determined using DTNB reagent according to Han et al. (12). Digested samples were combined with TGE buffer (0.086 mol/L Tris, 0.09 mol/L glycine, 4 mmol/L EDTA, pH 8.0). An aliquot of this mixture was then incubated with the DTNB reagent for 60 min at 25 °C in the absence of light. The formation of a yellow chromophore was measured spectrophotometrically at 412 nm. The SH group content was calculated based on the molar extinction coefficient of the 2-nitro-5-thiobenzoate anion (TNB^2−^), which is 13,600 L·mol^−1^·cm^−1^, and expressed as micromoles of SH per gram of lyophilized digesta (μmol/g).

### 2.5 Fourier-transform infrared spectroscopy (FTIR)

The secondary structure of the proteins was determined following the method of Qiu (13). The digested samples were initially pre-frozen at −80 °C for 1 h, subsequently freeze-dried in a lyophilizer for 48 h, and finally stored in a desiccator until analysis. Following digestion, spectral acquisition was performed using an FTIR spectrometer. To characterize the secondary structures of the proteins, curve fitting and double integration of the amide I band (1,700–1,600 cm^−1^) were conducted using OMNIC and PeakFit software. The spectral components corresponding to α-helix (1,660–1,650 cm^−1^), β-sheet (1,640–1,600 cm^1−^), β-turn (1,700–1,660 cm^−1^), and random coil (1,650–1,640 cm^−1^) were identified. The relative content of each secondary structure was represented by its respective peak area within this region.

### 2.6 Evaluation of protein digestibility

The protein content of the cooked beef before digestion (*W*0) was determined using the bicinchoninic acid (BCA) assay according to Lan et al. (2). Following the simulated intestinal digestion phase, the protein content remaining in the insoluble pellet (*W*1) was determined using the same analytical procedure. Protein digestibility, defined as the proportion of protein hydrolyzed during the *in vitro* gastrointestinal simulation, was calculated using the following equation:


$$\begin{array}{l} \text{Protein digestibility }\left(\text{\%}\right)=\left[\left(\text{W}0-\text{W}1\right)/\text{W}0\right]\times \;100\text{\%} \end{array}$$

### 2.7 Gel electrophoresis analysis

Protein fractions were extracted from the digestion products for electrophoretic analysis according to Sun et al. (14). Proteins present in the digestion supernatants were precipitated by adding four volumes of cold anhydrous ethanol and incubating at 4 °C overnight. The precipitated proteins were collected by centrifugation, redissolved in SDS-PAGE sample buffer (containing 2% SDS and 5% β-mercaptoethanol), and heated at 95 °C for 5 min. An equal amount of protein (20 μg) was loaded into each lane of a 12% polyacrylamide gel. Electrophoresis was conducted at constant voltages (90 V during the stacking phase and 120 V during the separation phase). Proteins were visualized by staining the gels with Coomassie Brilliant Blue R-250, and a pre-stained protein molecular weight marker was included for size reference.

### 2.8 Analysis of amino acids

The amino acid composition of the digestion products was determined using an automated amino acid analyzer (Hitachi L-8900, Japan) after acid hydrolysis. A 300 mg aliquot of each digested sample was mixed with 10 mL of 6 M HCl. The solution was flushed with nitrogen to expel air, sealed, and hydrolyzed in an oven at 110 °C for 22 h. The hydrolysate was then purged with nitrogen to remove residual air and filtered through a membrane. A 1 ml aliquot of the filtrate was concentrated to dryness under nitrogen, and the dried residue was redissolved in 1 ml of 0.02 M HCl and transferred into vials for analysis. Separation and quantification were achieved using a lithium citrate buffer system coupled with post-column ninhydrin detection. Individual amino acids were identified and quantified against external amino acid standards, and the contents were expressed as mg per liter of digesta (mg/L). Tryptophan was not determined due to its degradation under acid hydrolysis.

### 2.9 Measurement of antioxidant capacity

The antioxidant activity of the digestion samples was evaluated by measuring the radical scavenging capacity against ABTS^+^• and DPPH• radicals. The specific assay procedures were conducted according to Du et al. (15).

For ABTS^+^• scavenging activity, the ABTS^+^• radical cation was produced by reacting a 7 mM ABTS stock solution with 2.45 mM potassium persulfate and allowing the mixture to stand in the dark for 12–16 h. This stock solution was then diluted with 0.1 mol/L phosphate buffer (pH 7.4) to achieve an absorbance of 0.70 ± 0.02 at 734 nm. For the assay, 0.1 ml of the sample was mixed with 0.9 ml of the diluted ABTS^+^• working solution. After a 10-min incubation period in the dark, the absorbance was measured at 734 nm, with a blank prepared using distilled water.

For DPPH• scavenging assay, a 0.2 mmol/L DPPH solution was prepared in anhydrous ethanol. A 0.5 ml aliquot of the sample was combined with 1.2 ml of the DPPH solution, and the total volume was adjusted to 4.0 ml with distilled water. The mixture was vortexed and then incubated in darkness at room temperature for 30 min, after which its absorbance was read at 517 nm. A negative control, consisting of the sample mixed with ethanol instead of the DPPH solution, was prepared to account for any inherent sample color. The radical scavenging capacity (RSC) for both assays was quantified as a percentage using the formula: RSC (%) = [(A_control - A_sample) / A_control] × 100%.

### 2.10 Analysis of phenolics content and bioaccessibility

Phenolics were extracted from the freeze-dried digesta using an 80% methanol (v/v) solution via ultrasonication at 40 °C for 30 min. Following centrifugation, the supernatant was filtered through a 0.22 μm membrane and subjected to HPLC analysis coupled with a UV-Vis detector. Separation was performed on a reversed-phase C18 column (250 mm × 4.6 mm, 5 μm particle size) maintained at 30 °C, with gradient elution using water containing 0.1% formic acid (solvent A) and acetonitrile (solvent B) at a flow rate of 0.8 ml/min. Detection was carried out at 280 nm. Eight phenolics (gallic acid, *p*-hydroxybenzoic acid, chlorogenic acid, syringic acid, caffeic acid, *p*-coumaric acid, epicatechin and resveratrol) were identified by matching their retention times with those of certified reference standards, and quantified using external calibration curves constructed from at least five concentration levels (*R*^2^ > 0.99 for all analytes). Under identical chromatographic conditions, this method has been validated with determination coefficients (*R*^2^) of 0.9864–0.9992, limits of detection and quantification of 0.010–0.055 and 0.015–0.070 mg/L, and spiked recoveries of 96.2%−102.7% (*n* = 3).

The bioaccessibility of each phenolic in a given digestion phase was calculated as the percentage of its quantity detected in the digesta of that phase (gastric or gastrointestinal) relative to its total amount present in the initial wine-beef mixture prior to digestion.

### 2.11 Molecular docking

The amino acid sequence of bovine myosin was retrieved from the NCBI database (https://www.ncbi.nlm.nih.gov/). Homology modeling was performed using the Swiss-Model online server (https://swissmodel.expasy.org/) with 6YSY (sequence identity: 95.41%) as the structural template. The generated protein model was subsequently validated for geometric conformational rationality through Ramachandran plot analysis using the PROCHECK program within the SAVES v6.1 evaluation suite. The crystal structure of human pepsin (PDB ID: 1PSN) was obtained from the RCSB PDB database (https://www.rcsb.org/). Given its approximately 85% sequence identity and highly conserved catalytic center relative to the porcine pepsin used in the digestion experiments, this structure was selected as a structural surrogate for molecular docking.

The three-dimensional structures of gallic acid, caffeic acid, *p*-hydroxybenzoic acid, and *p*-coumaric acid were downloaded from the PubChem database (https://pubchem.ncbi.nlm.nih.gov/). These ligands were selected to represent the contrasting phenolic fates observed during co-digestion, as detailed in Section 3.3. To simulate the pH 3.0 environment characteristic of gastric digestion, the H++ server (http://newbiophysics.cs.vt.edu/H++/) was employed to adjust the protonation states of both pepsin and myosin, and Avogadro 1.2.0 software was utilized for charge processing and structural optimization of the four phenolic ligands under identical pH conditions. Molecular docking simulations were executed using AutoDock Vina 1.1.2 software to calculate and analyze the binding modes between pepsin and each phenolic ligand, as well as between myosin and each phenolic ligand. Based on the determined binding conformations of the small molecules, the HDOCK algorithm was applied to perform protein–protein docking simulations by treating the “pepsin-phenolics” complex as a whole with myosin. The intermolecular interactions of the generated complexes were comprehensively analyzed using LigPlot+ v2.3.1 software. All docking results and active pocket localizations were visualized using PyMOL™ 3.0.3 software. Finally, the binding free energy (Δ*G*) of the protein–protein complexes was quantitatively assessed using the PRODIGY online server to evaluate the predicted binding affinity among the components.

### 2.12 Statistical analysis

All experimental procedures were performed in triplicate, and results are expressed as the mean ± standard deviation. Data were analyzed using SPSS software (Version 23.0, IBM Corp.). For indices obtained from the factorial cooking × wine design (protein carbonyl content, free sulfhydryl content, protein digestibility and amino acid contents), two-way analysis of variance (ANOVA) was conducted separately for the gastric and gastrointestinal phases, with cooking method and co-digestion matrix (water or wine) as fixed factors. Main effects and the cooking × wine interaction were evaluated, and partial eta squared (partial η^2^) was calculated as an effect-size estimate. For wine-matrix indices that did not form a full factorial structure (ABTS and DPPH radical scavenging capacities and individual phenolic contents), one-way ANOVA was used to compare the meal-matrix groups separately within each digestion phase. When ANOVA indicated a significant effect, Duncan's multiple range test was used for *post-hoc* comparisons. A *p*-value < 0.05 was considered statistically significant.

## 3 Results and discussion

### 3.1 Impact of mixed meal on the digestive behavior of beef proteins

#### 3.1.1 Protein oxidation

To evaluate the combined effects of cooking technique and wine co-digestion on protein oxidation, the concentrations of carbonyl and free sulfhydryl (SH) groups were determined. Two-way ANOVA showed significant main effects of cooking method and wine co-digestion, together with a significant cooking × wine interaction, for carbonyl content in both phases (gastric interaction, *p* = 0.015; gastrointestinal interaction, *p* < 0.001). As shown in Figures 1a, b, the carbonyl content in the beef + water groups (Control I) was significantly influenced by the cooking method, and grilled beef (GTC) exhibited the highest carbonyl content, markedly greater than that of steamed (STC) and boiled (BTC) beef. The higher level in GTC may be attributable to thermal oxidative stress induced by dry-heat and high-temperature conditions, which promote direct protein carbonylation, as well as the Maillard reaction that generates carbonyl-containing compounds capable of associating with proteins (16, 17). Notably, co-digestion with wine led to a significant inhibition of carbonyl formation across all cooking methods. Relative to their corresponding water controls, the reductions during the gastric phase were 21.5%, 30.8%, and 22.7% for STW, BTW, and GTW, respectively; in the intestinal phase, reductions of 47.8%, 45.3%, and 23.2% were observed.

**Figure 1:**
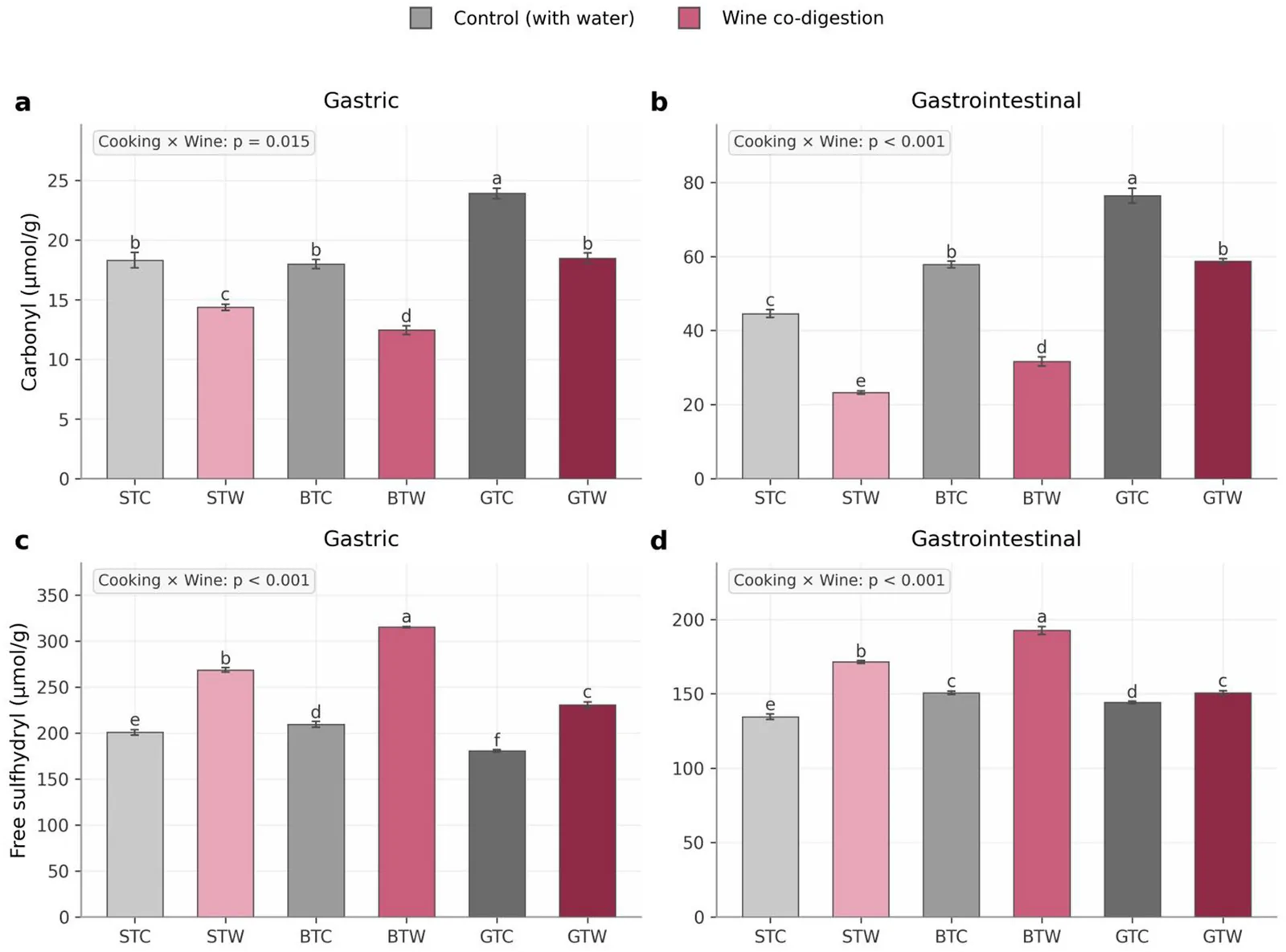
Protein carbonyl **(a, b)** and free sulfhydryl **(c, d)** contents of cooked beef after simulated gastric **(a, c)** and gastrointestinal **(b, d)** digestion, without or with red wine co-digestion. Data are presented as mean ± SD (*n* = 3). Different lowercase letters within each panel indicate significant differences among samples (two-way ANOVA followed by Duncan's multiple range test, *p* < 0.05).

As illustrated in Figures 1c, d, the free SH content in STC, BTC, and GTC during the gastric phase was 200.9, 209.6, and 180.5 μmol/g, respectively. Two-way ANOVA revealed significant main effects of cooking method and wine co-digestion and a significant cooking × wine interaction for free SH content in both digestive phases (all *p* < 0.001). The lower value in GTC is consistent with the notion that intense heat from grilling generates more ROS, which can oxidize free SH groups and promote disulfide bond formation. Co-digestion with wine effectively preserved these free SH groups. Specifically, concurrent wine consumption increased the free SH content during the gastric phase by 33.7%, 50.5%, and 27.7% for STW, BTW, and GTW, respectively, with corresponding increases of 27.4%, 27.9%, and 4.4% in the intestinal phase. This protective effect may be attributed to phenolic compounds acting as radical scavengers, neutralizing ROS and thereby alleviating the oxidative conversion of free SH groups to disulfide bonds (18). The extent of protection varied with cooking method, with the most pronounced effects observed in boiled samples.

Collectively, these results indicate that the inhibitory effect of wine phenolics on protein oxidation is context-dependent, varying according to both the cooking-induced oxidative state and the digestive phase. A plausible explanation is that phenolics in wine scavenge reactive oxygen species (ROS) generated during digestion, thereby protecting amino acid side chains from oxidation into carbonyl groups (19).

Moreover, it was noted that regardless of cooking method or the presence of wine, carbonyl content increased by an average of 2.4-fold from the gastric to the intestinal phase, while free SH content decreased by an average of 1.6-fold. These phase-dependent changes may be explained by trypsin hydrolysis exposing additional oxidation-sensitive sites, continuous attack by reactive oxygen species, and the alkaline intestinal environment that weakens phenol-protein complexes and increases protein susceptibility to oxidation (20). Furthermore, quinones formed from phenolic oxidation irreversibly deplete free sulfhydryl groups via Michael addition or thiol-quinone coupling (21), while proteolytic unfolding exposes buried thiols to disulfide bond formation (2).

#### 3.1.2 Protein secondary structure

To evaluate the effects of wine co-digestion and cooking techniques on protein secondary structure, FTIR was employed (Supplementary Figure S1). During the gastric phase, the amide I band (1,700–1,600 cm^−1^) exhibited distinct peak positions depending on the cooking method. In the control groups, the absorption peaks for STC, BTC and GTC were located at 1,658.1, 1,650.7 and 1,658.2 cm^−1^, respectively; while in the experimental groups, the corresponding peaks were observed at 1,661.9, 1,658.2 and 1,660.1 cm^−1^ for STW, BTW and GTW, respectively. The experimental groups exhibited a noticeable blue shift of the amide I band relative to the controls, consistent with the possibility that phenolics contributed to the stabilization of protein conformation through hydrogen bonding or hydrophobic interactions, thereby partially counteracting structural alterations induced by thermal processing (22). During the intestinal phase, the absorption peaks for STC, BTC and GTC were located at 1,678.8, 1,680.2, and 1,673.1 cm^−1^, respectively; in the experimental groups, the peaks shifted to 1,683.3, 1,682.6, and 1,684.5 cm^−1^, respectively. The experimental groups again exhibited noticeable blue shifts relative to their corresponding controls.

The secondary structure distribution was further analyzed by deconvolution and second-derivative fitting of the amide I band (Figure 2). In the control groups, steaming and boiling resulted in a higher relative content of α-helices and a lower content of β-sheets compared to grilling, which exhibited the highest proportion of random coils. Specifically, during the intestinal phase, STC and BTC showed 3.8 and 3.2 percentage points higher α-helix content than GTC, respectively, while β-sheet content was reduced by 0.4 and 1.6 percentage points, respectively. These differences may be explained by the distinct heat transfer mechanisms and chemical environments of each cooking method. Steaming and boiling employ moist heat at relatively mild temperatures, which induces controlled protein denaturation with limited aggregation. Under these conditions, hydrogen bonds within α-helices are partially preserved, and the ordered structure is largely maintained. In contrast, grilling involves high-temperature dry heat and rapid heat transfer, which severely disrupts hydrogen bonds, causes extensive unfolding of α-helices, and promotes their conversion into β-sheet structures. Additionally, the dry-heat environment facilitates oxidative cross-linking and Maillard reactions, further stabilizing aggregated conformations and increasing random coil content (23). Consequently, grilled beef proteins adopt a more disordered and aggregated state, which is consistent with their lower α-helix and higher β-sheet/random coil proportions.

**Figure 2:**
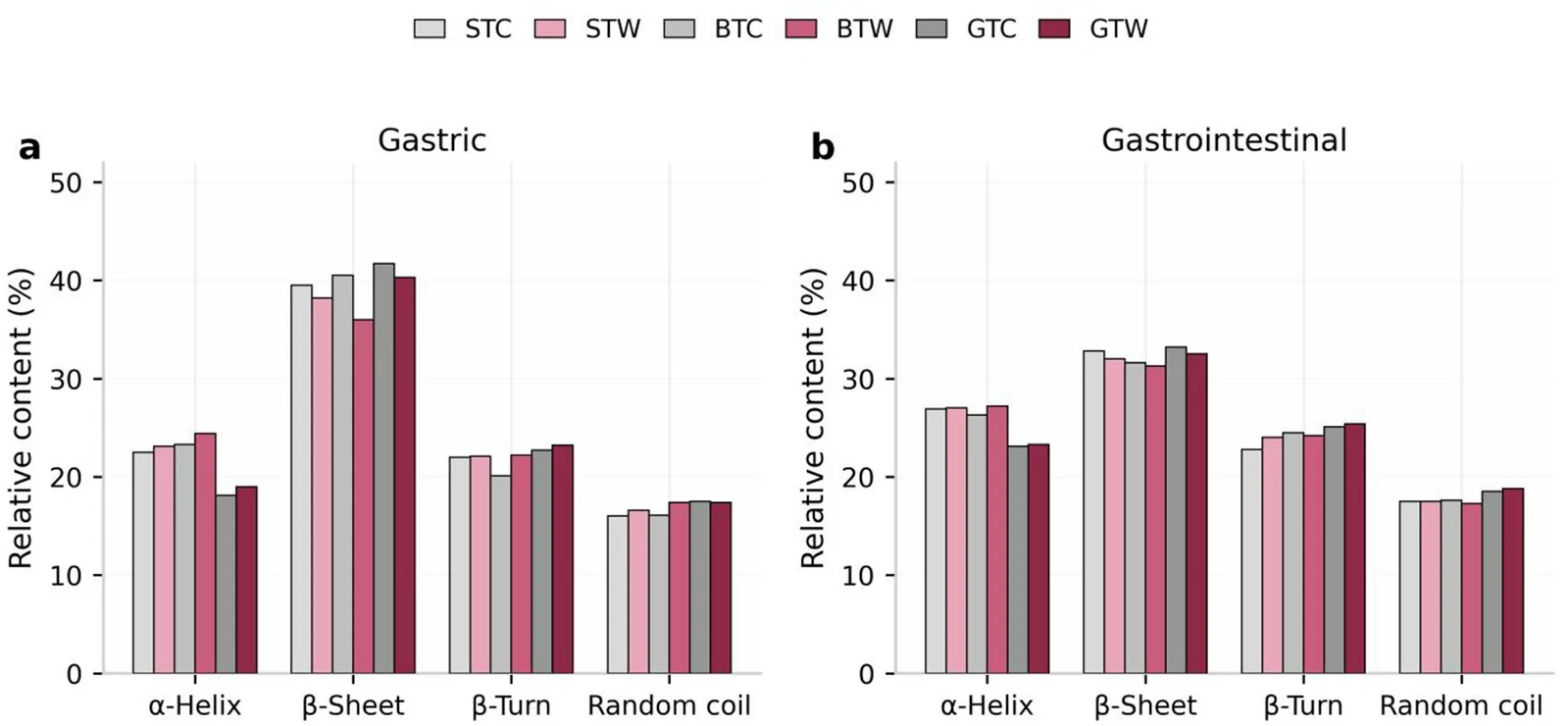
Relative contents of protein secondary structures of cooked beef after simulated gastric **(a)** and gastrointestinal **(b)** digestion, determined by deconvolution of the amide I band (1,600–1,700 cm^−1^) in FTIR spectra. Values are expressed as mean (*n* = 3).

In the experimental groups (STW, BTW and GTW), the secondary-structure pattern was consistent with a protective role of wine phenolics. Across the gastric and intestinal phases, the average α-helix content in the three experimental groups was 0.6 percentage points (2.8% relative) higher than in the corresponding control groups, while the average β-sheet content was 1.5 percentage points (3.9% relative) lower. These findings suggest that wine phenolics may have helped to preserve the hydrogen bonding network within protein backbones through non-covalent interactions, stabilizing native-like conformations and limiting excessive heat-induced unfolding that would otherwise convert α-helices into β-sheets or random coils. Furthermore, by scavenging ROS generated during thermal processing and digestion, wine phenolics may have alleviated oxidative damage that would otherwise promote protein aggregation and secondary structure disruption. The apparent protective pattern was most evident in the boiled samples during the gastric phase (α-helix +1.1 percentage points; β-sheet −4.5 percentage points), suggesting that phenolics can partially compensate for the structural alterations induced by cooking.

#### 3.1.3 Protein digestibility

In the gastric phase (Figure 3a), two-way ANOVA revealed significant main effects of cooking method and wine co-digestion on protein digestibility (*p* < 0.001), whereas their interaction was not significant (*p* = 0.165). GTC exhibited the lowest digestibility (36.4%) among the control groups, whereas STC and BTC achieved substantially higher values (46.4% and 44.6%, respectively). This observation may be attributed to the distinct molecular consequences of thermal processing, where steaming and boiling induced controlled denaturation under mild conditions that limited excessive aggregation, while grilling promoted oxidative cross-linking and protein aggregation through disulfide bonds and other covalent modifications, thus hindering enzyme-substrate interactions (24, 25).

**Figure 3:**
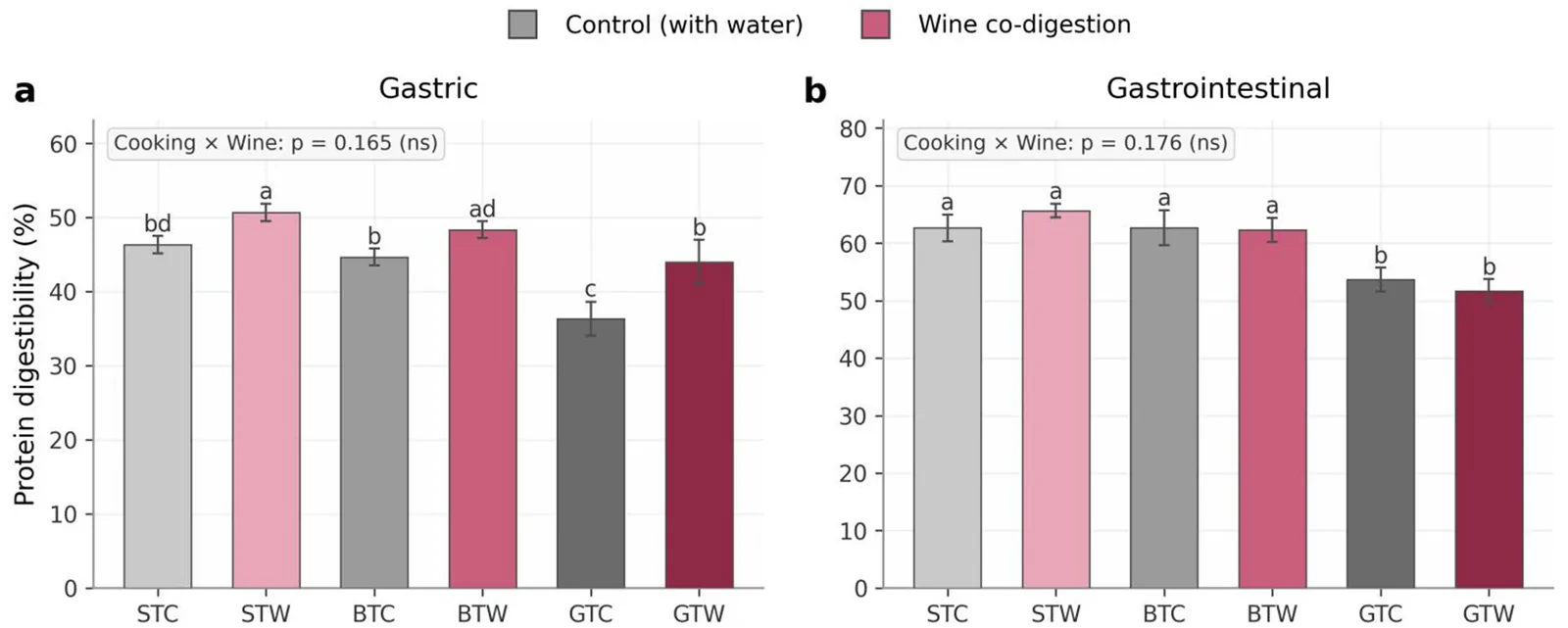
*In vitro* protein digestibility of cooked beef after simulated gastric **(a)** and gastrointestinal **(b)** digestion. Data are presented as mean ± SD (*n* = 3). Different lowercase letters within each panel indicate significant differences among samples (two-way ANOVA followed by Duncan's multiple range test, *p* < 0.05).

When co-digested with wine, gastric digestibility was markedly enhanced across all cooking methods. Relative to their corresponding controls, digestibility increased by 8.9%, 8.6% and 20.9% for STW, BTW and GTW, respectively. The observed improvement could be ascribed to the possible dual role of wine phenolics. On one hand, by scavenging ROS, phenolics alleviated protein oxidation and thereby preserved structural integrity amenable to proteolytic cleavage. On the other hand, phenolics could induce moderate structural unfolding through non-covalent interactions, exposing additional cleavage sites for pepsin. The numerical increase was greatest in grilled beef, suggesting that wine co-digestion partially counteracted the negative impact of intense thermal processing (19).

Upon entering the intestinal phase, the overall digestibility increased across all samples (by 7.7–18.1 percentage points relative to the gastric phase). However, the advantage conferred by wine co-digestion during the gastric phase did not persist into the intestinal phase: the digestibility of STW remained comparable to its control (+5.2%), whereas that of BTW and GTW was slightly lower than their corresponding controls (−0.5% and −4.1%, respectively), with no significant difference between the control and wine-co-digested samples within any cooking method (Figure 3b). Consistent with this pattern, two-way ANOVA showed no significant wine main effect (*p* = 0.834) or cooking × wine interaction (*p* = 0.176) in the gastrointestinal phase. This observation can be explained by the dynamic changes in phenol-protein interactions: during the gastric phase, non-covalent interactions exerted protective and unfolding effects (22), whereas in the intestinal environment these interactions were weakened by the alkaline pH, which may have led to the dissociation of the proposed ternary complex and the re-association of phenolics with newly generated peptide fragments, potentially altering their accessibility to proteases. Consistent with the digestibility data, SDS-PAGE profiles showed an attenuation of the ~55 kDa myosin band in the wine-co-digested groups during the gastric phase, with nearly complete disappearance of intact protein bands after intestinal digestion (Supplementary Figure S2).

#### 3.1.4 Amino acid composition of digestion products

To further characterize the proteolytic products released during co-digestion, the amino acid composition of the digestion products in the experimental groups and controls was determined throughout gastrointestinal digestion. A total of 16 amino acids were identified, including 8 essential and 8 non-essential amino acids, with their contents shown in Figure 4 and Supplementary Table S1.

**Figure 4:**
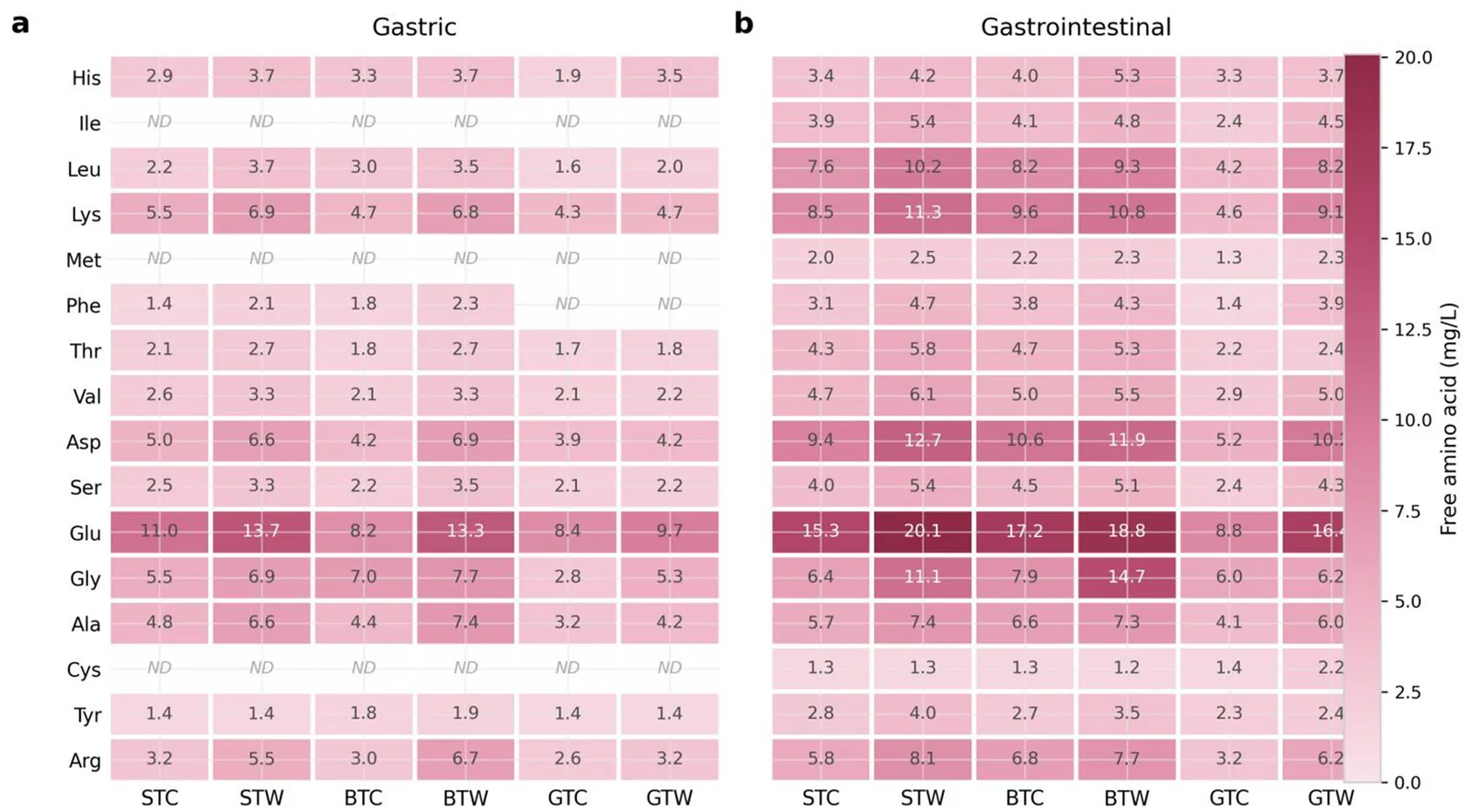
Heatmap of amino acid contents (mg/L) of cooked beef after simulated gastric **(a)** and gastrointestinal **(b)** digestion. Values are expressed as mean (*n* = 3). ND, not detected. Two-way ANOVA was performed separately for each digestion phase.

Two-way ANOVA of the gastric phase data revealed that total, essential and non-essential amino acid contents were significantly affected by cooking method, wine co-digestion and their interaction (all *p* < 0.001). The total amino acid contents in STC and BTC were significantly higher than those in GTC. When wine was co-digested with beef, the total contents in STW and BTW were 49.9% and 57.4% higher than that in GTW, respectively. Specifically, compared with the water controls, the essential amino acid content in STW, BTW, and GTW increased by 33.7%, 34.0%, and 22.5%, respectively, while the non-essential amino acid content increased by 32.1%, 53.6%, and 23.4%, respectively.

A similar factorial pattern was observed in the intestinal phase, with significant main effects of both factors and significant cooking × wine interactions for all three amino acid categories (all *p* < 0.001). As digestion progressed into the intestinal phase, the amino acid content in each group increased by an average of 1.8-fold compared with the gastric phase. In the mixed-meal groups, the total amino acid contents in STW, BTW and GTW reached 120.2, 117.7 and 92.8 mg/L, respectively. Among these, GTW showed the largest numerical increase relative to its matched water control (67.2%), a pattern consistent with a stronger association between wine co-digestion and amino acid accumulation under the highest initial protein oxidation. Because the wine matrix itself contributed amino acids, this increase should not be attributed exclusively to enhanced beef proteolysis. Notably, isoleucine, methionine and cysteine were detected only during the intestinal phase, suggesting that peptides carrying these residues became soluble and accessible predominantly in the intestinal environment.

The consistently higher amino acid content in wine-co-digested groups compared with Control I across both gastric and intestinal phases reflects the combined contribution of beef-derived proteolysis and amino acids or small peptides inherently present in the wine matrix. Accordingly, these increases should not be interpreted solely as enhanced digestion of beef protein. Several non-exclusive mechanisms may contribute to the observed pattern. First, the wine matrix provided its own amino acids and small peptides, which served as direct components of the measured amino acid pool and as potential substrates for digestive enzymes. Second, wine phenolics may have induced moderate structural unfolding of beef proteins through non-covalent interactions, exposing additional cleavage sites for pepsin and trypsin. This effect was numerically most pronounced in GTW, where the total amino acid concentrations increased by 23.1% and 67.2% in the gastric and intestinal phases, respectively. Third, the antioxidant activity of phenolics may have protected oxidation-sensitive amino acid residues (such as arginine, lysine, tyrosine and cysteine) from oxidative damage, thereby limiting aggregation and cleavage-site masking, which could be particularly relevant for grilled samples. Fourth, in the intestinal phase, the phenolics–protein complexes proposed for the gastric phase may have been partially disrupted, releasing previously protected cleavage sites and allowing free phenolics to interact with new peptide fragments. This intestinal-specific unmasking hypothesis was consistent with the exclusive intestinal detection of isoleucine, methionine and cysteine; however, the residue-specific increases were not uniform, as intestinal isoleucine was higher in all three wine-co-digested groups, methionine was higher in STW and GTW, and cysteine was higher only in GTW relative to their matched controls.

### 3.2 Effect of mixed meal on the digestive behavior of wine phenolics

#### 3.2.1 Antioxidant activity

The antioxidant activity of the digesta was evaluated using DPPH and ABTS radical scavenging assays (Figure 5). One-way ANOVA revealed significant meal-matrix effects on ABTS and DPPH radical scavenging capacity (RSC) in the gastric phase (both *p* < 0.001) and the gastrointestinal phase (ABTS, *p* < 0.001; DPPH, *p* = 0.037). Co-digestion of beef with wine resulted in higher antioxidant activity than wine alone (Control II), indicating that the mixed-meal matrix yielded a higher RSC than wine phenolics alone, consistent with the combined contributions of wine phenolics and beef-derived antioxidants.

**Figure 5:**
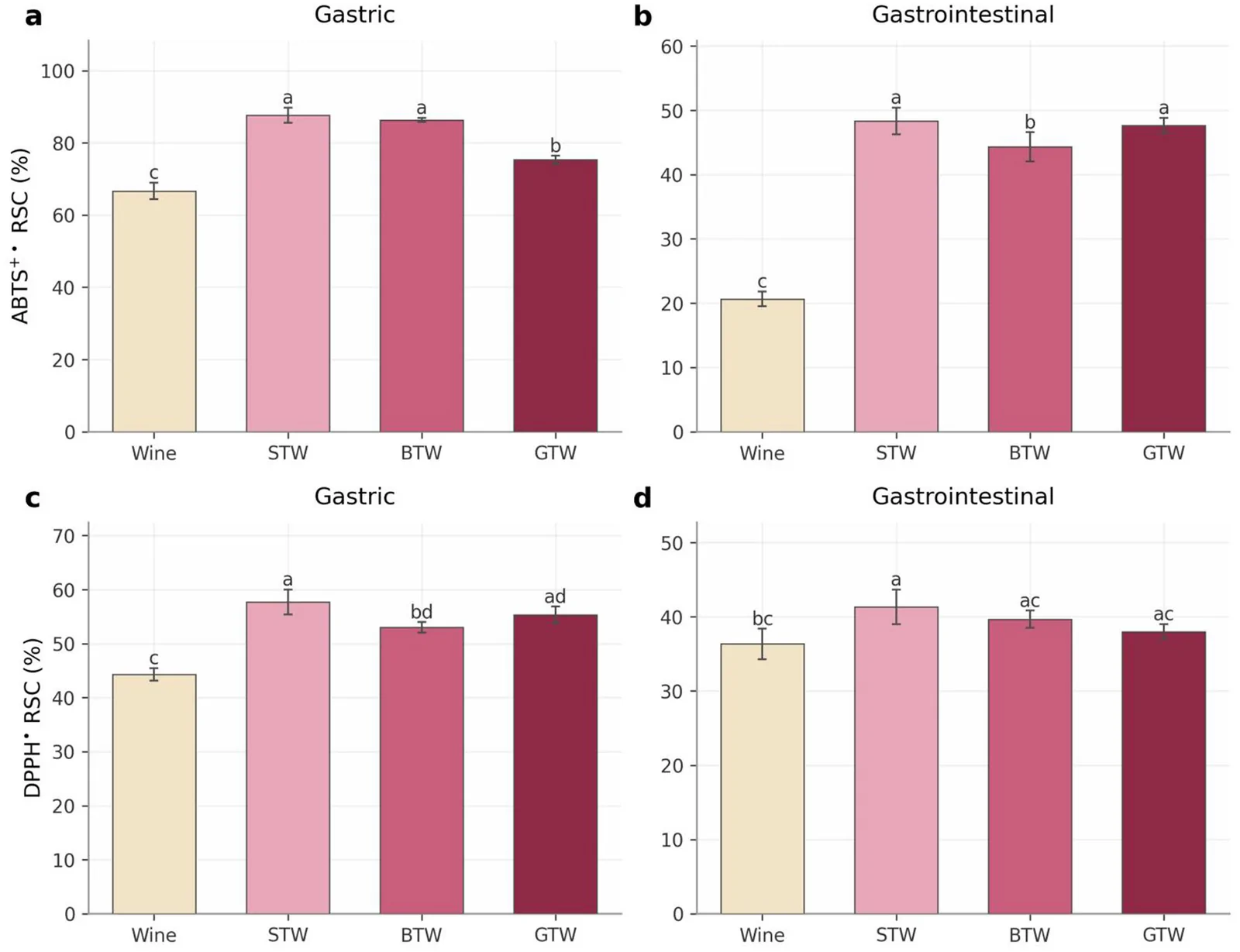
Radical scavenging capacity of the digesta against ABTS^+^• **(a, b)** and DPPH• **(c, d)** after simulated gastric **(a, c)** and gastrointestinal **(b, d)** digestion. Data are presented as mean ± SD (*n* = 3). Different lowercase letters within each panel indicate significant differences among samples (one-way ANOVA followed by Duncan's multiple range test, *p* < 0.05).

During the gastric phase, the mixed digesta exhibited average increases of 24.9% in ABTS and 24.5% in DPPH RSC relative to wine alone. In the intestinal phase, the average increases were 125.3% for ABTS and 9.1% for DPPH. Notably, the mixed digesta exhibited a greater scavenging capacity against ABTS^+^• radicals than against DPPH•. Such difference could be due to the broader sensitivity of the ABTS assay to both hydrophilic and lipophilic antioxidants, which allows for a more comprehensive assessment of the diverse antioxidant species present in the complex mixed-meal digesta (26).

The higher antioxidant effect observed in the mixed-meal matrix could be explained by three reasons. First, the radical scavenging activity of phenolics was complemented by beef-derived components. Hydrogen atoms or electrons donated by phenolics such as gallic and p-coumaric acids neutralized free radicals (DPPH• or ABTS+•), thereby interrupting oxidative chain reactions that could otherwise damage amino acid side chains and protein backbones. Within the mixed-meal context, this activity was further supported by antioxidant peptides or amino acids released from beef during digestion, resulting in enhanced overall capacity. Second, the metal-chelating effect of phenolics was particularly relevant in the presence of the beef matrix. Transition metals such as iron and copper, which catalyze Fenton-type reactions, may be chelated by these phenolic compounds. This chelation inhibited the generation of highly reactive hydroxyl radicals, which are responsible for critical oxidative events including cysteine thiol oxidation and protein carbonylation (27). Third, the binding of phenolics to proteins contributed to the stabilization of protein secondary structures, and may inhibit excessive unfolding and aggregation, thereby helping to maintain sulfhydryl group content (28).

Additionally, it was noted that the RSC decreased after intestinal digestion compared to the gastric phase, with average reductions of 49.7% (ABTS) and 25.8% (DPPH). This decline could be attributed to the alkaline pH of the intestinal environment, which could alter the stability and reactivity of phenolic compounds. Additionally, interactions between phenolics and digestive enzymes or digestion by-products, as well as potential degradation of phenolics under intestinal conditions, could contribute to the observed reduction in antioxidant activity.

#### 3.2.2 Simulated digestion of wine phenolics

The concentrations and bioaccessibility of eight phenolics were monitored throughout simulated digestion (Figure 6). For these wine-matrix indices, statistical comparisons were performed among wine alone, STW, BTW and GTW using one-way ANOVA. Co-digestion of beef and wine resulted in notable alterations in phenolic profiles compared to Control II, reflecting the impact of the mixed-meal matrix on phenolic stability and release.

**Figure 6:**
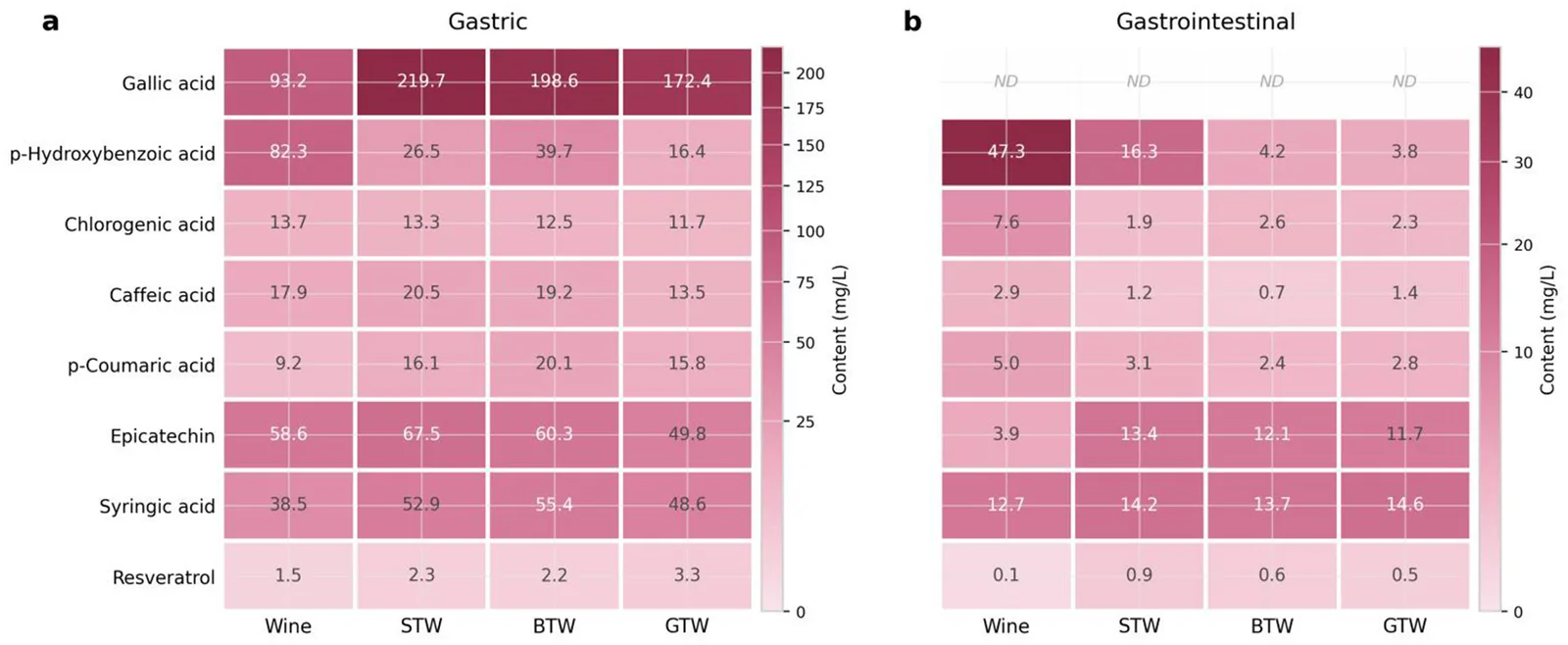
Heatmap of phenolic contents (mg/L) after simulated gastric **(a)** and gastrointestinal **(b)** digestion. Values are expressed as mean (*n* = 3). ND, not detected. The color scale was square-root transformed to improve visualization of compounds; statistical analyses were performed on untransformed concentrations.

In the gastric phase, the concentration changes were consistent with the bioaccessibility pattern. One-way ANOVA indicated significant meal-matrix effects for all quantified phenolics except chlorogenic acid (*p* = 0.090; all others *p* < 0.001). Gallic acid, *p*-coumaric acid and resveratrol showed higher contents in the mixed-meal groups than in Control II (e.g., gallic acid increased by 135.7%, 113.1% and 84.9% in STW, BTW and GTW, respectively), an enhancement that may be partly attributable to the hydrolysis of galloylated flavanols and hydrolysable tannins during gastric digestion, as well as to the protective effect of the beef matrix against phenolic degradation (29). At the bioaccessibility level (Supplementary Figure S3a), the co-digestion system markedly improved the gastric availability of most phenolics: *p*-coumaric acid bioaccessibility nearly doubled (from 30.9% to 54.0%−67.8%), and that of syringic acid and gallic acid increased by 17.3–27.5 and 19.7–31.6 percentage points, respectively. In contrast, the bioaccessibility of *p*-hydroxybenzoic acid dropped sharply from 82.7% to 16.5%−39.8%, consistent with its preferential capture by the protein matrix.

Upon entering the intestinal phase, the contents of most phenolics declined rapidly. Significant meal-matrix effects were observed for *p*-hydroxybenzoic acid, chlorogenic acid, caffeic acid, *p*-coumaric acid, epicatechin and resveratrol (all *p* < 0.001), whereas the effect for syringic acid was not significant (*p* = 0.215). Gallic acid was undetectable in all groups, indicating its instability under alkaline conditions or its conversion into oxidation/polymerization products and protein adducts. *p*-Hydroxybenzoic acid, chlorogenic acid, *p*-coumaric acid and caffeic acid also decreased markedly relative to the gastric phase; accordingly, their intestinal bioaccessibility in the mixed-meal groups fell to low levels (3.8%−16.9% for *p*-hydroxybenzoic acid, 7.9%−9.7% for chlorogenic acid, 2.3%−4.9% for caffeic acid, and 7.8%−10.4% for *p*-coumaric acid; Supplementary Figure S3b), all below those of the wine-only control. Such declines probably resulted from the alkaline pH of the simulated environment, the activity of pancreatic enzymes, and continual protein-phenolic interactions (30).

Two phenolics showed substantial increases in the intestinal phase. The concentrations of epicatechin in STW, BTW and GTW were 3.48, 3.13 and 3.04 times those of Control II, respectively, and resveratrol levels were 7.2–11.6 times those of Control II. This enrichment was mirrored at the bioaccessibility level, where epicatechin rose from 4.8% in wine alone to 14.7%−16.6% in the mixed-meal groups, and resveratrol from 0.8% to 5.2%−9.3%. These observations may be explained by the dynamic dissociation of the proposed gastric ternary complexes. During the gastric phase, these phenolics were embedded in the phenolics-pepsin-myosin complex through non-covalent interactions, which may have limited free degradation. Upon entering the intestinal phase, hydrogen bonds and hydrophobic interactions were weakened by the alkaline environment, and the complex may have been disrupted by trypsin-mediated cleavage of the protein scaffold, potentially leading to the release of previously sequestered phenolics. Meanwhile, newly exposed peptide fragments provided alternative binding sites for phenolics, which could form soluble peptide–phenolic aggregates that further enhanced the apparent bioaccessibility (31).

Overall, the bioaccessibility trends were consistent with the concentration variations, suggesting that phenolic bioaccessibility may be selectively regulated by the co-digestion system rather than simply following phenolic abundance. The preferential gastric retention and subsequent intestinal release of epicatechin and resveratrol, together with the reduced bioaccessibility of *p*-hydroxybenzoic acid and other highly polar phenolics, suggest that differential protein-phenolic interactions may contribute to their distinct digestive fates. These observations raise the possibility that protein-enzyme-phenolic association acts as a selectivity filter during co-digestion, a hypothesis examined in the molecular docking analysis presented in Section 3.3. Such matrix-dependent regulation of phenolic bioaccessibility has likewise been observed in other plant-derived systems subjected to simulated gastrointestinal digestion (32, 33).

### 3.3 Molecular docking

Molecular docking was employed to predict the binding modes and affinities among phenolics, pepsin and beef proteins at the atomic level, with the aim of interpreting two gastric-phase observations from the co-digestion experiments: the protection and enhanced digestibility of beef protein in the presence of wine (vs. Control I), and the improved bioaccessibility of wine phenolics in the presence of beef (vs. Control II).

Myosin, the predominant myofibrillar protein in beef skeletal muscle (approximately 55% of the total), is also the primary substrate for pepsin-mediated proteolysis during gastric digestion. Following Fu et al. (34), who docked phenolics against a native myofibrillar model at the digestion stage, the myosin heavy chain (MHC) was selected as the modeling target to elucidate protein–phenolic interactions within the beef–wine co-digestion matrix. Three representative phenolics were then selected to cover the three distinct fates observed during gastric co-digestion: gallic acid (markedly increased), caffeic acid (moderately increased) and *p*-hydroxybenzoic acid (reduced). Their binding interactions with myosin and pepsin, together with the formation of phenolics-pepsin-myosin ternary complexes, were simulated to generate a molecular-level hypothesis for the observed co-digestion effects.

#### 3.3.1 Molecular docking of phenolics with myosin

A three-dimensional model of bovine myosin heavy chain was first constructed via homology modeling using SWISS-MODEL (Supplementary Figure S4) and used as the receptor for docking. Supplementary Figure S5 illustrates the predicted binding sites and interacting residues of the three phenolics within the myosin cavity. All three compounds were predicted to bind favorably with negative binding energies, following the order gallic acid (−6.8 kcal/mol) > caffeic acid (−6.5 kcal/mol) > p-hydroxybenzoic acid (−5.5 kcal/mol) (Supplementary Figures S5a, c, e). Their predicted binding poses within the hydrophobic pocket suggested anchoring via hydrogen bonds with key residues (Supplementary Figures S5b, d, f), which may constrain local conformational fluctuations, thereby stabilizing myosin structure while simultaneously protecting the phenolics from rapid oxidative degradation.

The docking results provided a predicted molecular-level rationale for two interrelated protective mechanisms that could operate in the beef-wine co-digestion matrix. First, the predicted binding of phenolics to myosin is consistent with stabilization of the protein structure against oxidative attack, which aligns with the reduced protein oxidation (e.g., lower carbonyl formation and preserved sulfhydryl groups) in the wine-co-digested groups compared to Control I. Second, the predicted formation of non-covalent complexes may physically protect the phenolics from rapid oxidation into quinones during gastric digestion, thereby preserving their bioaccessibility. Notably, the predicted binding-energy hierarchy was concordant with the bioaccessibility data reported in Section 3.2.2: the strongest predicted binding affinity (gallic acid) corresponded to the highest bioaccessibility enhancement, while the weakest predicted binding (*p*-hydroxybenzoic acid) was associated with reduced bioaccessibility. These concordant results are consistent with the hypothesis that stronger protein-binding affinity may confer greater phenolic stability and improved bioaccessibility during co-digestion.

#### 3.3.2 Molecular docking of phenolics with pepsin

Among the eight phenolics quantified during gastric digestion, gallic acid and *p*-coumaric acid, which showed the most pronounced gastric-content increases, were docked against pepsin. Both were predicted to bind with appreciable affinity (−5.4 and −5.5 kcal/mol, respectively), and neither occupied the catalytic dyad (Asp32–Asp215). Gallic acid was predicted to be anchored at the rim of the active pocket (Supplementary Figures S6a, b), and *p*-coumaric acid by a combination of hydrogen bonds and hydrophobic contacts (Supplementary Figures S6c, d). The predicted binding outside the catalytic center may restrict conformational flexibility of adjacent residues, thereby potentially stabilizing the active conformation of pepsin while preserving its catalytic activity.

To examine how phenolic binding may affect the pepsin–myosin interaction, ternary complexes were then constructed *in silico* (Figure 7). The binding energies of myosin with free pepsin, with the gallic acid/pepsin complex, and with the *p*-coumaric acid/pepsin complex were −10.9, −15.6 and −13.9 kcal/mol, respectively, suggesting that phenolic binding may enhance the affinity between pepsin and its substrate. This interpretation is further supported by analysis of the predicted enzyme–substrate interfaces (Figures 7d–f). When pepsin was docked to myosin alone, the interface exhibited only a few long-range hydrogen bonds (2.4–3.3 Å) and sparse van der Waals contacts (Figure 7d). In contrast, the gallic acid/pepsin complex formed several short, strong hydrogen bonds (2.1–2.2 Å) along with more than 15 pairs of van der Waals contacts (Figure 7e); the *p*-coumaric acid/pepsin complex likewise formed a predicted hydrogen-bond network with myosin (Figure 7f). Notably, in both ternary systems the phenolic ligand was situated at the predicted pepsin–myosin interface and engaged in intermolecular contacts (Figures 7e, f), suggesting that the phenolics may act as bridging scaffolds that stabilize the enzyme–substrate association beyond mere allosteric modulation.

**Figure 7:**
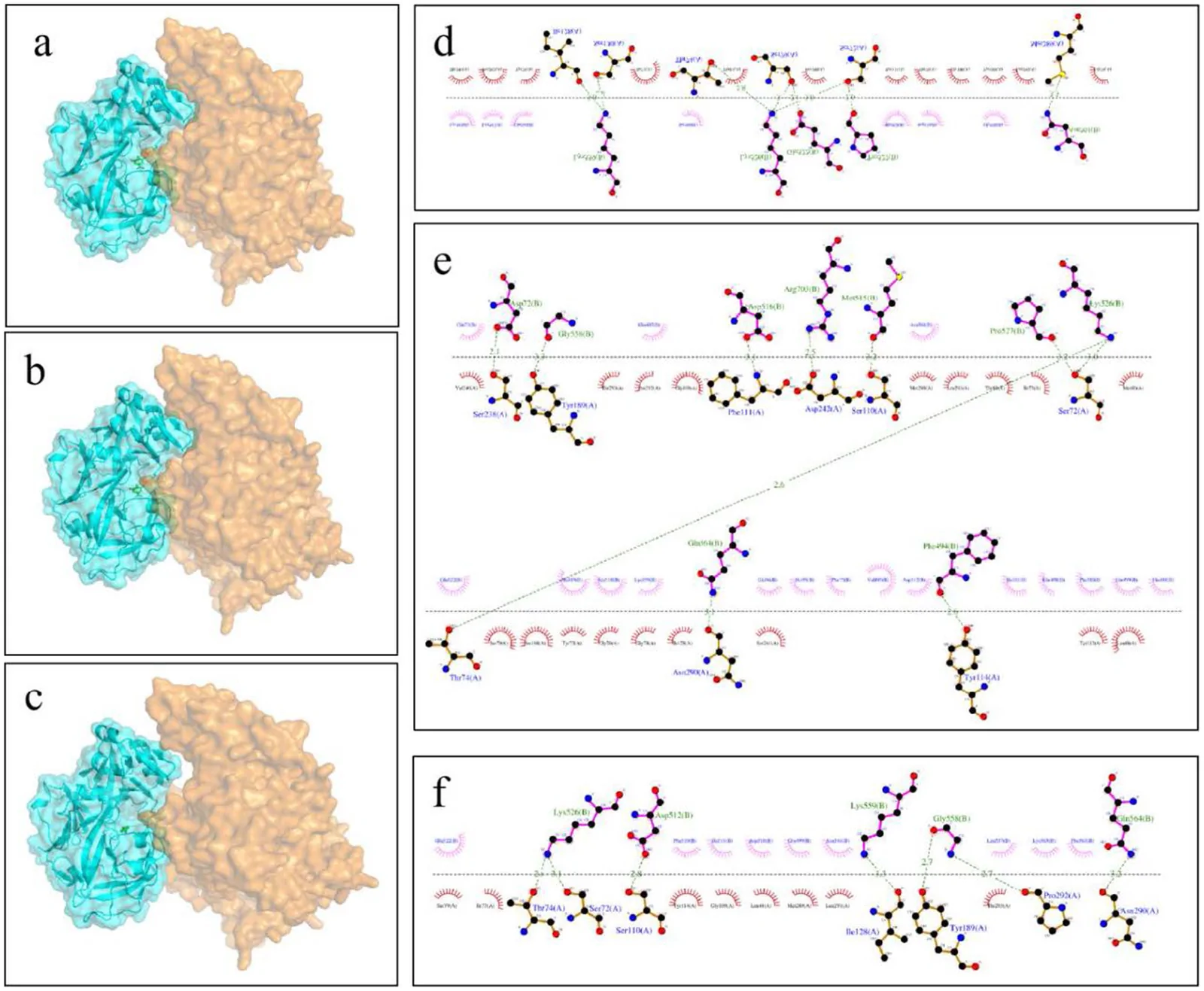
Molecular docking results of phenolics, pepsin and myosin. Three-dimensional conformations of pepsin/myosin **(a)**, gallic acid/pepsin/myosin complex **(b)**, and *p*-coumaric acid/pepsin/myosin complex **(c)**. Two-dimensional interaction diagrams of the residues involved in molecular docking: pepsin /myosin **(d)**, gallic acid/pepsin/myosin complex **(e)**, and *p*-coumaric acid/pepsin/ myosin complex **(f)**.

Collectively, these docking results provide a plausible molecular rationale for the enhanced gastric digestibility observed in the mixed-meal groups: phenolic binding outside the catalytic dyad may preserve pepsin catalytic function while stabilizing the pepsin-myosin interface, thereby facilitating pepsin-mediated proteolysis.

However, these interpretations should be considered within several methodological constraints. First, docking was confined to the gastric phase because pepsin is the principal gastric protease and the digestibility differences were most evident at this stage; intestinal docking was not performed because extensive myosin hydrolysis precluded the construction of a reliable structural model. Second, the homology model was built from native myosin rather than from cooking-specific oxidized or aggregated forms. Constructing such tailored models would require experimentally resolved structural data followed by customized molecular dynamics simulations, an approach that is currently impractical. Third, human pepsin was used as a structural surrogate for the porcine enzyme employed in the digestion experiments, and molecular docking cannot by itself establish the formation of a stable ternary complex or demonstrate enhanced pepsin activity in the digesta. The predicted binding modes and their proposed effects on enzyme-substrate association should therefore be regarded as hypotheses requiring validation through biophysical binding assays and pepsin-kinetic measurements.

### 3.4 Integrated model of reciprocal modulation in the beef–wine co-digestion matrix

The concept of reciprocal modulation proposed in this study aims to reveal the asymmetric coupling between proteins and phenolics in a mixed meal. Unlike traditional studies that often view phenolics merely as protein protectors or treat proteins solely as phenolic carriers, the present study integrates both perspectives. Therefore, we propose two conceptual models corresponding to the two digestive phases: a gastric-phase model centered on the predicted formation and function of a ternary complex, and an intestinal-phase model describing the possible dissociation of this complex and the subsequent redistribution of released phenolics. Together, these two models provide a dynamic working framework for interpreting the beef–wine co-digestion matrix.

#### 3.4.1 Gastric-phase model: the ternary complex as a functional hub

By integrating our present findings, a mechanistic model for the gastric phase is proposed (Figure 8). In this model, wine phenolics, pepsin, and the myosin heavy chain are hypothesized to form a ternary complex, whose formation is predicted by molecular docking and supported indirectly by concordant biochemical results. Rather than being a simple physical aggregate, this proposed complex may function as a molecular hub with bidirectional regulatory capacity, enabling simultaneous protein protection and phenolic stabilization.

**Figure 8:**
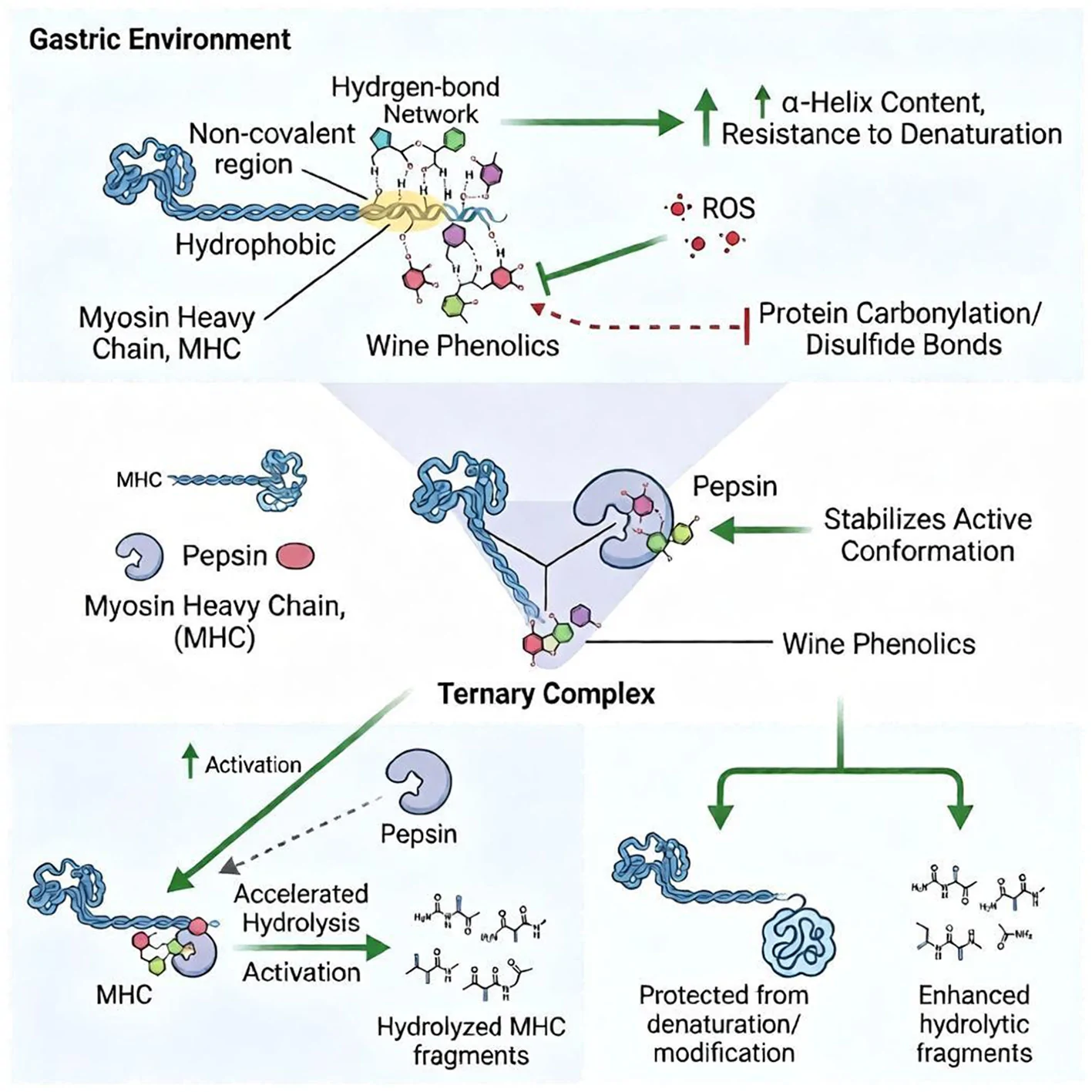
Proposed model of ternary complex-mediated reciprocal modulation in the beef–wine co-digestion matrix during the gastric phase.

With respect to protein protection, incorporation of phenolics into the hydrophobic cavity of MHC may restrict local conformational fluctuations through hydrogen-bond networks. Concurrently, the radical-scavenging capacity of phenolics may neutralize gastric reactive oxygen species, limiting carbonyl formation and sulfhydryl oxidation. These dual effects of structural constraint and chemical protection, synergistically preserve myosin against heat and oxidation induced damage. The outcome is most evident in the grilled beef co-digested with wine, where digestibility and amino acid release increased by 20.9% and 67.2%, respectively, despite the highest initial oxidative load.

Regarding phenolic stabilization, physical sequestration within the protein–enzyme matrix may shield reactive hydroxyl groups from rapid gastric oxidation, thereby enhancing bioaccessibility. The binding-energy hierarchy (gallic acid > caffeic acid > *p*-hydroxybenzoic acid) was consistent with the bioaccessibility ranking observed experimentally, supporting the hypothesis that the retention efficiency of each phenolic may be related to the stability of its proposed ternary complex. For *p*-hydroxybenzoic acid, its weaker predicted binding affinity, combined with its low molecular weight and high polarity, may favor association with oxidatively modified protein aggregates or Maillard products, leading to a marked reduction in bioaccessibility.

In terms of enzymatic regulation, docking predicts that phenolics may bind to pepsin outside its catalytic dyad and stabilize its active conformation. The phenolics–pepsin complex exhibits markedly higher affinity for MHC than free pepsin, resulting in a more stable enzyme–substrate complex and potentially accelerated proteolysis. Thus, within the ternary complex, phenolics act simultaneously as protein stabilizers and allosteric facilitators of pepsin activity, and this dual functionality explains both the structural protection and the enhanced proteolysis observed experimentally.

#### 3.4.2 Intestinal-phase model: dissociation and redistribution

Although molecular docking was confined to gastric conditions, the simulated digestive data obtained in the gastrointestinal environment were used to develop a kinetic projection model (Supplementary Figure S7). In this model, the proposed ternary complex is hypothesized to undergo pH-driven dissociation upon entering the intestinal phase. Three charge-related changes may jointly disrupt the complex: deprotonation of phenolic hydroxyls reduces hydrogen-bond donor capacity; negative charging of protein side chains (glutamic acid and aspartic acid) enhances electrostatic repulsion; and alkaline inactivation of pepsin removes its enzymatic anchoring function from the complex.

Following dissociation, released phenolics may diverge into two competing pathways. Pathway A, favored by catechol-containing phenolics (e.g., epicatechin) and lipophilic compounds (e.g., resveratrol), may lead to reassociation with tryptic peptides or incorporation into bile salt micelles, resulting in enhanced intestinal bioaccessibility. Pathway B, which may prevail for phenolics such as *p*-hydroxybenzoic acid that formed strong hydrophobic or melanoidin-like associations in the gastric phase, may result in entrapment within insoluble protein residues, thereby reducing their bioaccessibility.

The release of amino acids is temporally coupled with phenolic redistribution. Isoleucine, methionine and cysteine, detected only in the intestinal phase, showed residue-specific responses: intestinal isoleucine was significantly elevated in all three co-digestion groups, methionine was higher in STW and GTW, and cysteine was higher only in GTW relative to their matched controls. These patterns are consistent with the hypothesis that trypsin hydrolysis exposes new cleavage sites as the proposed ternary complex disassembles. Concurrently, the reassociation of phenolics with nascent peptide fragments may alter peptide solubility and enzyme accessibility, potentially creating a positive feedback loop that couples proteolysis with phenolic release.

In summary, the proposed model suggests asymmetric coupling within the beef–wine co-digestion matrix. Phenolics appeared to protect proteins through antioxidant activity and conformational stabilization, thereby promoting protein digestion. Reciprocally, proteins appeared to preserve phenolics by gastric sequestration and to enable their controlled release in the intestine, thereby enhancing bioaccessibility. The proposed ternary complex may serve as the structural scaffold for this bidirectional coupling, exhibiting a non-additive property that cannot be simply predicted from separate binary interactions. This putative synergy highlights the potentially emergent nature of component interactions and provides a mechanistic framework for the nutritional optimization of protein–phenolic co-digestion.

While the proposed model provides a coherent framework for interpreting the reciprocal responses observed in the beef–wine co-digestion matrix, several limitations should be addressed here. First, because no ethanol-matched control was included, the potential contribution of ethanol to the observed effects cannot be excluded. Under the dilution conditions used here, the ethanol concentration was estimated to be approximately 2% (v/v) in the gastric phase and 1% (v/v) in the intestinal phase; at these levels, ethanol may influence protein solubility, enzyme activity or phenolic partitioning in addition to the effects of phenolics. Second, the static *in vitro* model cannot reproduce dynamic gastric emptying, intestinal transit, absorption, or host and microbial metabolism. Third, only one red wine was tested, limiting the generalizability of the findings to wines with different phenolic profiles, ethanol contents or matrix compositions. Finally, the proposed ternary-complex model is based partly on predictive molecular docking; its formation and functional consequences require further validation through biophysical, enzymatic and *in vivo* studies.

## 4 Conclusion

This study combined *in vitro* gastrointestinal digestion with molecular docking to investigate the bidirectional modulation between beef proteins and wine phenolics in a mixed meal. The results support a proposed phenolics–protease–myosin ternary complex as a plausible molecular basis for this reciprocity, benefiting both components simultaneously. For proteins, wine phenolics appeared to act as protectors: by binding to myosin and pepsin within the proposed ternary complex, they may stabilize myosin secondary structure against oxidative attack while concurrently enhancing its digestibility. This effect was most pronounced in grilled beef, where the highest initial oxidative load was associated with the greatest increases in gastric digestibility and intestinal amino acid content. For phenolics, the beef matrix appeared to serve as a carrier: the proposed ternary complex may function as a gastric reservoir that sequesters phenolics during the gastric phase and enables their controlled, pH-dependent release in the intestinal phase, selectively elevating the bioaccessibility of epicatechin and resveratrol. Collectively, these findings support the ternary complex as a shared molecular hub that may orchestrate reciprocal modulation in mixed meals. Future work should include ethanol-matched controls, biophysical and enzyme-kinetic validation of the proposed complex, dynamic digestion models, and *in vivo* studies to confirm these mechanisms and guide the optimization of cooking methods and meal pairing strategies.

## Data Availability

The original contributions presented in the study are included in the article/Supplementary material, further inquiries can be directed to the corresponding author.
