# Supplementary material for: Reciprocal modulation between beef protein and wine phenolics during in vitro co-digestion: a proposed ternary complex-mediated mechanism modulated by cooking technique
Source: Front Nutr. 2026 Oct 14;13:1967708. doi: 10.3389/fnut.2026.1967708 (PMC13714533; doi:10.3389/fnut.2026.1967708)
Supplement: Supplementary file 1 [file Data_Sheet_1.pdf]

Supplementary Material

Table S1. Statistical groupings of amino acid contents (mg/L) in the gastric and gastrointestinal digesta.

Fig.S1 Fourier-transform infrared (FTIR) spectra of cooked beef after simulated gastric and gastrointestinal digestion. (a) Steamed beef; (b) boiled beef; (c) grilled beef.

Fig.S2 SDS-PAGE of proteins of steak samples after simulated gastric (a) and gastrointestinal (b) digestion.

Fig. S3 Heatmap of the bioaccessibility (%) of wine phenolic compounds after simulated gastric (a) and gastrointestinal (b) digestion. ND, not detected. Values are expressed as mean (n = 3).

Fig. S4 (a) Three-dimensional structure of bovine myosin heavy chain. (b) Ramachandran plot of the bovine myosin heavy chain model.

Fig.S5 Molecular docking results of three representative phenolics with MHC: three-dimensional conformations of gallic acid (a), caffeic acid (c), and *p*-hydroxybenzoic acid (e); magnified three-dimensional views of the binding sites for gallic acid (b), caffeic acid (d), and *p*-hydroxybenzoic acid (f).

Fig.S6 Molecular docking results of two representative phenolics with pepsin: three-dimensional conformations of gallic acid (a) and *p*-coumaric acid (c); magnified three-dimensional views of the binding sites for gallic acid (b) and *p*-coumaric acid (d).

Fig.S7 Proposed model of ternary complex dissociation and phenolic redistribution in the beef–wine co-digestion matrix during the intestinal phase.

Table S1. Statistical groupings of amino acid contents (mg/L) in the gastric and gastrointestinal digesta.

Amino acid	Phase	STC	STW	BTC	BTW	GTC	GTW	*p* (Cooking)	*p* (Wine)	*p* (Cooking × Wine)
Histidine	G	2.92 ± 0.01 d	3.71 ± 0.07 a	3.33 ± 0.05 c	3.74 ± 0.00 a	1.87 ± 0.03 e	3.52 ± 0.18 b	< 0.001	< 0.001	< 0.001
	GI	ND	ND	ND	ND	ND	ND			
Leucine	G	2.25 ± 0.03 c	3.69 ± 0.04 a	3.01 ± 0.17 b	3.53 ± 0.36 a	1.63 ± 0.09 d	2.04 ± 0.02 c	< 0.001	< 0.001	< 0.001
	GI	5.46 ± 0.05 b	6.93 ± 0.06 a	4.67 ± 0.06 c	6.79 ± 0.48 a	4.33 ± 0.73 c	4.67 ± 0.06 c	< 0.001	< 0.001	0
Methionine	G	ND	ND	ND	ND	ND	ND			
	GI	1.45 ± 0.22 c	2.10 ± 0.01 ad	1.79 ± 0.03 bd	2.27 ± 0.28 a	ND	ND	0	< 0.001	0
Threonine	G	2.13 ± 0.02 b	2.74 ± 0.02 a	1.78 ± 0.00 c	2.74 ± 0.15 a	1.74 ± 0.24 c	1.84 ± 0.02 c	< 0.001	< 0.001	< 0.001
	GI	2.60 ± 0.02 b	3.31 ± 0.02 a	2.13 ± 0.01 c	3.33 ± 0.20 a	2.05 ± 0.33 c	2.18 ± 0.03 c	< 0.001	< 0.001	< 0.001
Aspartic acid	G	4.98 ± 0.04 b	6.59 ± 0.06 a	4.19 ± 0.01 c	6.92 ± 0.43 a	3.87 ± 0.61 c	4.17 ± 0.04 c	< 0.001	< 0.001	< 0.001
	GI	2.55 ± 0.02 c	3.32 ± 0.02 b	2.25 ± 0.01 d	3.55 ± 0.15 a	2.08 ± 0.22 d	2.16 ± 0.01 d	< 0.001	< 0.001	< 0.001
Glutamic acid	G	10.97 ± 0.10 b	13.73 ± 0.08 a	8.18 ± 0.02 d	13.34 ± 0.89 a	8.38 ± 0.25 d	9.66 ± 0.11 c	< 0.001	< 0.001	< 0.001
	GI	5.48 ± 0.02 c	6.87 ± 0.02 b	7.04 ± 0.05 b	7.65 ± 0.02 a	2.84 ± 0.01 d	5.32 ± 0.23 c	< 0.001	< 0.001	< 0.001
Alanine	G	4.79 ± 0.04 c	6.63 ± 0.02 b	4.44 ± 0.01 d	7.37 ± 0.31 a	3.22 ± 0.01 e	4.22 ± 0.02 d	< 0.001	< 0.001	< 0.001
	GI	ND	ND	ND	ND	ND	ND			
Tyrosine	G	1.37 ± 0.02 b	1.35 ± 0.02 b	1.79 ± 0.02 a	1.89 ± 0.38 a	1.39 ± 0.10 b	1.39 ± 0.01 b	< 0.001	0	0
	GI	3.18 ± 0.06 c	5.53 ± 0.09 b	2.99 ± 0.06 ce	6.72 ± 0.61 a	2.64 ± 0.06 de	3.18 ± 0.02 c	< 0.001	< 0.001	< 0.001
Total essential amino acids (EAA)	G	16.81 ± 0.27 b	22.49 ± 0.09 a	16.71 ± 0.16 b	22.40 ± 0.40 a	11.62 ± 0.87 d	14.25 ± 0.12 c	< 0.001	< 0.001	< 0.001
	GI	33.32 ± 0.12 c	44.03 ± 0.02 b	30.87 ± 0.10 d	47.44 ± 1.83 a	24.42 ± 0.64 e	30.10 ± 0.25 d	< 0.001	< 0.001	< 0.001
Total amino acids	G	50.13 ± 0.38 c	66.51 ± 0.11 b	47.58 ± 0.16 d	69.84 ± 1.74 a	36.04 ± 0.23 f	44.35 ± 0.28 e	< 0.001	< 0.001	< 0.001
	GI	3.44 ± 0.03 c	4.16 ± 0.02 b	3.99 ± 0.01 bd	5.34 ± 0.33 a	3.35 ± 0.40 c	3.69 ± 0.04 cd	< 0.001	< 0.001	0
Isoleucine	G	3.88 ± 0.03 e	5.40 ± 0.01 a	4.12 ± 0.01 d	4.79 ± 0.04 b	2.38 ± 0.02 f	4.45 ± 0.22 c	< 0.001	< 0.001	< 0.001
	GI	7.65 ± 0.03 d	10.16 ± 0.01 a	8.25 ± 0.03 c	9.28 ± 0.01 b	4.18 ± 0.02 e	8.18 ± 0.32 c	< 0.001	< 0.001	< 0.001
Lysine	G	8.49 ± 0.02 e	11.32 ± 0.02 a	9.57 ± 0.05 c	10.76 ± 0.00 b	4.62 ± 0.04 f	9.13 ± 0.38 d	< 0.001	< 0.001	< 0.001
	GI	1.98 ± 0.01 c	2.48 ± 0.03 a	2.25 ± 0.01 b	2.27 ± 0.01 b	1.27 ± 0.04 d	2.26 ± 0.10 b	< 0.001	< 0.001	< 0.001

Phenylalanine	G	3.07 ± 0.01 ^{d}	4.66 ± 0.03 ^{a}	3.76 ± 0.02 ^{c}	4.25 ± 0.01 ^{b}	1.42 ± 0.40 ^{e}	3.86 ± 0.15 ^{c}	< 0.001	< 0.001	< 0.001
	GI	4.32 ± 0.02 ^{d}	5.78 ± 0.00 ^{a}	4.73 ± 0.02 ^{c}	5.35 ± 0.00 ^{b}	2.20 ± 0.02 ^{f}	2.38 ± 0.10 ^{e}	< 0.001	< 0.001	< 0.001
Valine	G	4.67 ± 0.01 ^{d}	6.06 ± 0.01 ^{a}	4.98 ± 0.02 ^{c}	5.53 ± 0.01 ^{b}	2.88 ± 0.05 ^{e}	5.00 ± 0.19 ^{c}	< 0.001	< 0.001	< 0.001
	GI	9.44 ± 0.05 ^{e}	12.67 ± 0.01 ^{a}	10.63 ± 0.05 ^{c}	11.87 ± 0.01 ^{b}	5.19 ± 0.03 ^{f}	10.17 ± 0.40 ^{d}	< 0.001	< 0.001	< 0.001
Serine	G	4.02 ± 0.00 ^{e}	5.42 ± 0.00 ^{a}	4.45 ± 0.02 ^{c}	5.07 ± 0.00 ^{b}	2.35 ± 0.00 ^{f}	4.32 ± 0.16 ^{d}	< 0.001	< 0.001	< 0.001
	GI	15.31 ± 0.04 ^{e}	20.08 ± 0.03 ^{a}	17.17 ± 0.08 ^{c}	18.82 ± 0.00 ^{b}	8.79 ± 0.05 ^{f}	16.45 ± 0.63 ^{d}	< 0.001	< 0.001	< 0.001
Glycine	G	6.44 ± 0.03 ^{d}	11.14 ± 0.02 ^{b}	7.86 ± 0.03 ^{c}	14.65 ± 0.40 ^{a}	5.98 ± 0.49 ^{d}	6.17 ± 0.01 ^{d}	< 0.001	< 0.001	< 0.001
	GI	5.66 ± 0.02 ^{c}	7.41 ± 0.01 ^{a}	6.59 ± 0.03 ^{b}	7.34 ± 0.00 ^{a}	4.06 ± 0.42 ^{d}	5.96 ± 0.23 ^{c}	< 0.001	< 0.001	< 0.001
Cysteine	G	1.32 ± 0.01 ^{cf}	1.29 ± 0.00 ^{df}	1.33 ± 0.01 ^{c}	1.25 ± 0.01 ^{e}	1.37 ± 0.04 ^{b}	2.25 ± 0.01 ^{a}	< 0.001	< 0.001	< 0.001
	GI	2.82 ± 0.01 ^{c}	4.01 ± 0.00 ^{a}	2.73 ± 0.01 ^{d}	3.49 ± 0.01 ^{b}	2.27 ± 0.04 ^{f}	2.38 ± 0.10 ^{e}	< 0.001	< 0.001	< 0.001
Arginine	G	5.85 ± 0.07 ^{e}	8.12 ± 0.04 ^{a}	6.76 ± 0.05 ^{c}	7.70 ± 0.06 ^{b}	3.16 ± 0.05 ^{f}	6.16 ± 0.29 ^{d}	< 0.001	< 0.001	< 0.001
	GI	37.50 ± 0.07 ^{e}	50.03 ± 0.03 ^{a}	41.65 ± 0.03 ^{c}	47.57 ± 0.33 ^{b}	22.30 ± 0.81 ^{f}	38.95 ± 1.00 ^{d}	< 0.001	< 0.001	< 0.001
Total non-essential amino acids (NEAA)	G	50.86 ± 0.03 ^{d}	70.13 ± 0.08 ^{a}	57.52 ± 0.17 ^{b}	70.19 ± 0.40 ^{a}	33.17 ± 0.64 ^{e}	53.86 ± 1.20 ^{c}	< 0.001	< 0.001	< 0.001
	GI	88.36 ± 0.09 ^{e}	120.16 ± 0.06 ^{a}	99.17 ± 0.20 ^{c}	117.76 ± 0.38 ^{b}	55.47 ± 1.31 ^{f}	92.82 ± 2.00 ^{d}	< 0.001	< 0.001	< 0.001

Values are expressed as mean ± SD (n = 3). Different lowercase letters within the same row indicate significant differences among the six samples (two-way ANOVA followed by Duncan's multiple range test, $p < 0.05$). ND, not detected. *p*-values are given for the main effects of cooking method and wine co-digestion and their interaction (two-way ANOVA).

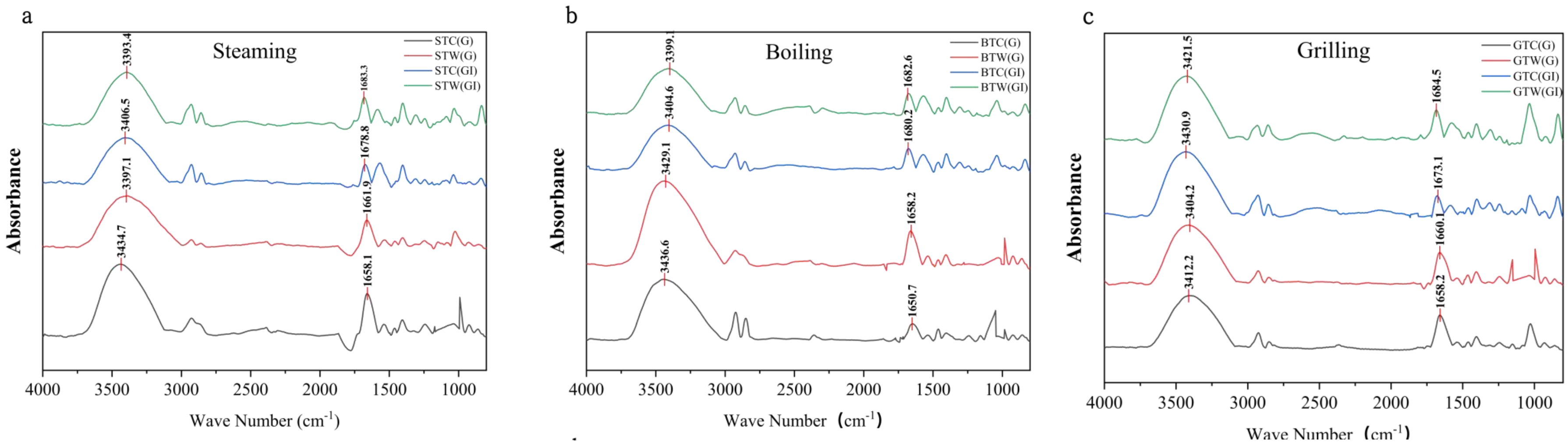

Fig.S1 Fourier-transform infrared (FTIR) spectra of cooked beef after simulated gastric and gastrointestinal digestion. (a) Steamed beef; (b) boiled beef; (c) grilled beef.

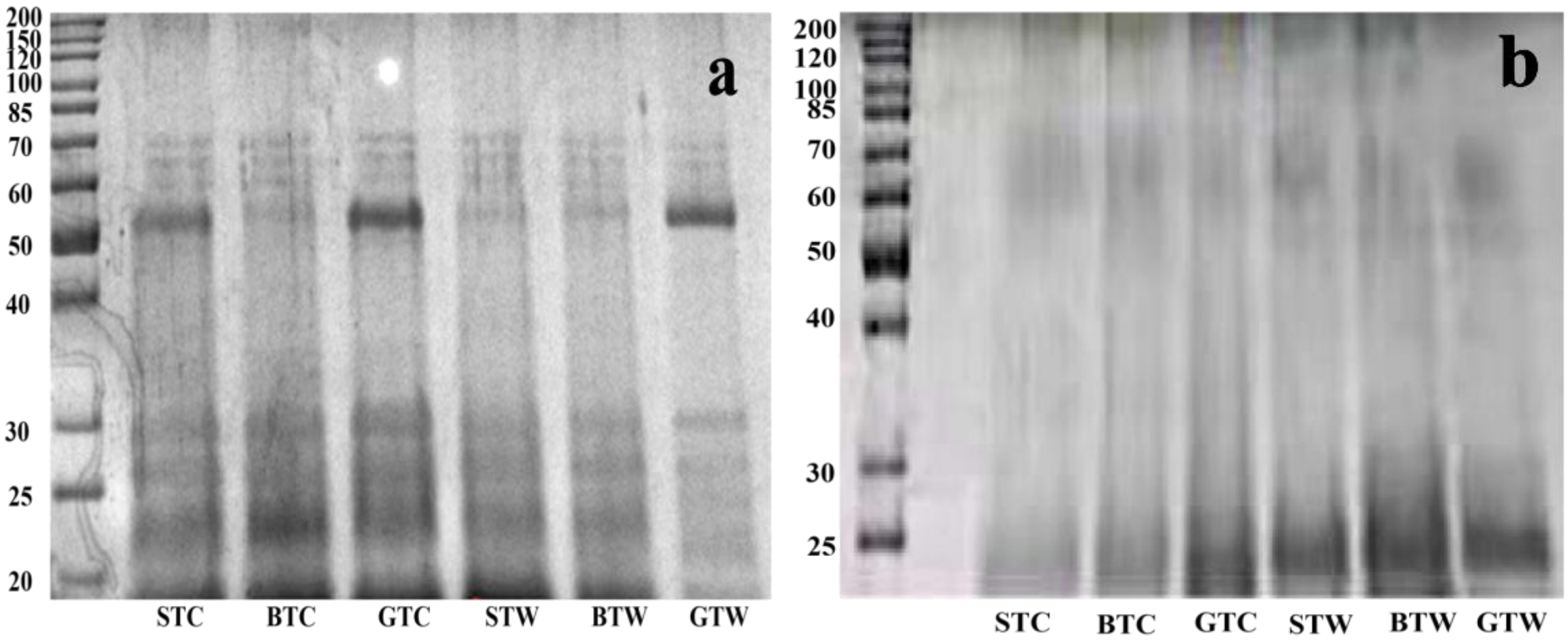

Fig.S2 SDS-PAGE of proteins of steak samples after simulated gastric (a) and gastrointestinal (b) digestion.

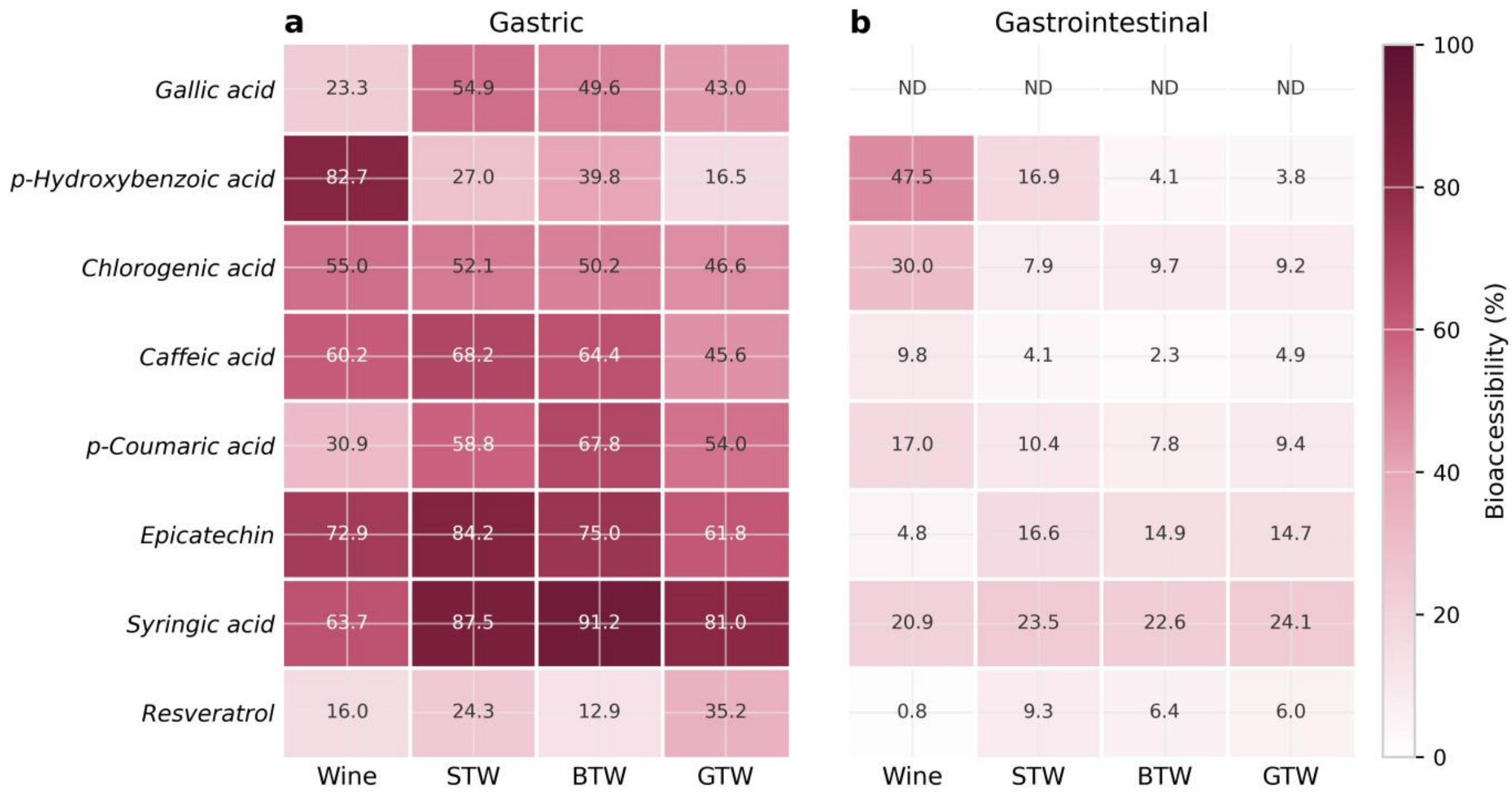

Fig. S3 Heatmap of the bioaccessibility (%) of wine phenolic compounds after simulated gastric (a) and gastrointestinal (b) digestion. ND, not detected. Values are expressed as mean (n = 3).

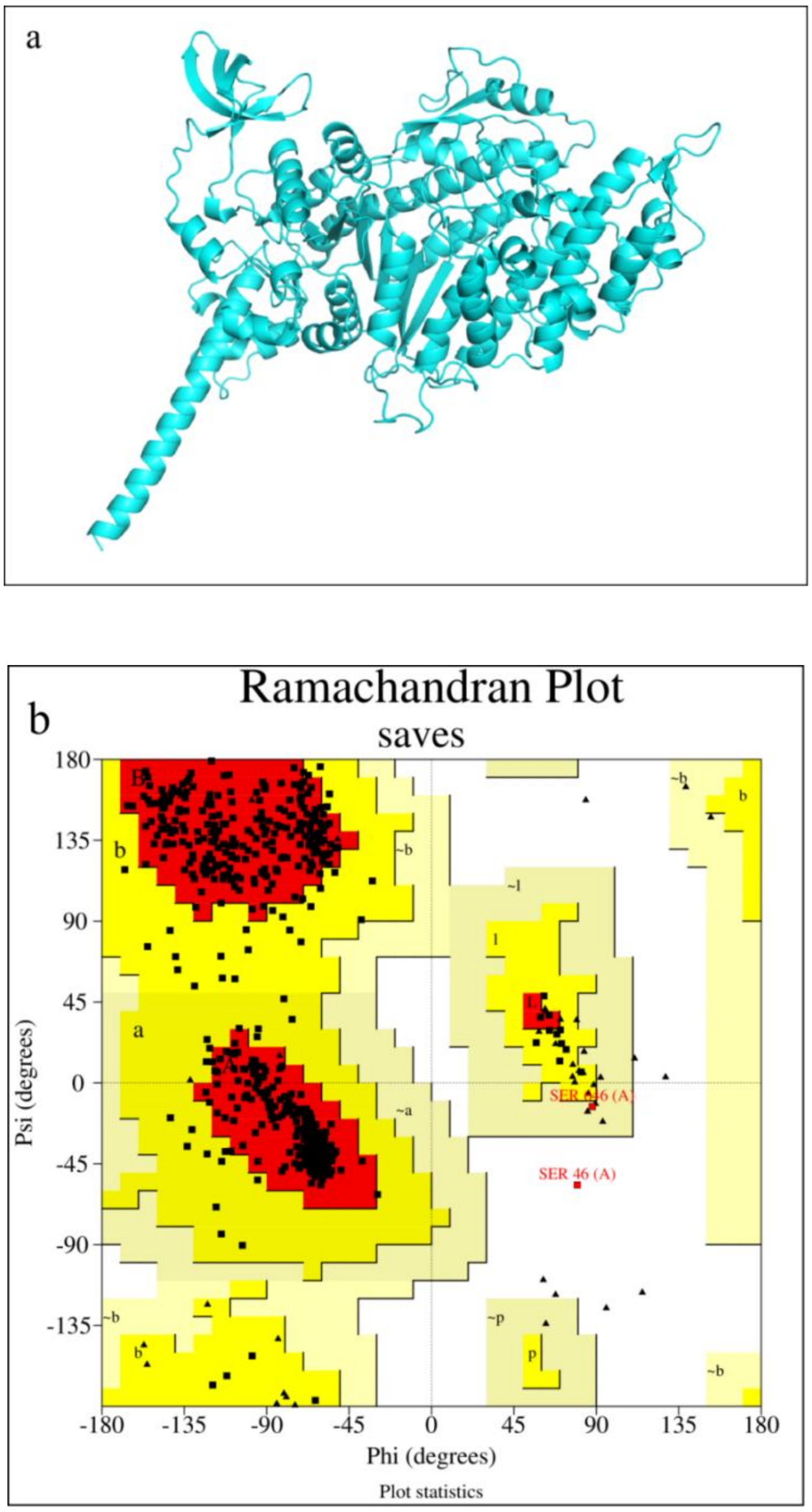

Fig. S4 (a) Three-dimensional structure of bovine myosin heavy chain. (b) Ramachandran plot of the bovine myosin heavy chain model.

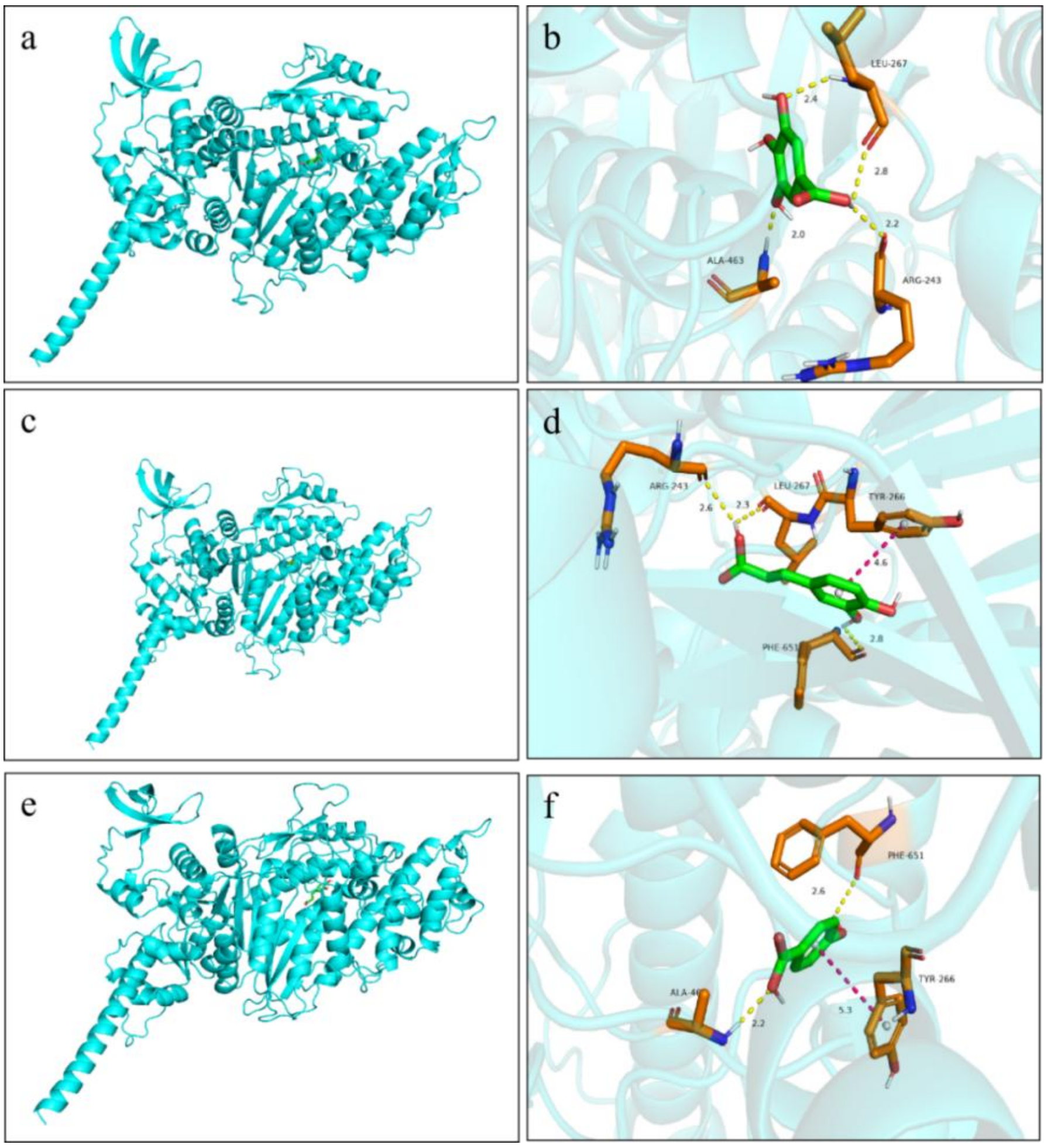

Fig.S5 Molecular docking results of three representative phenolics with MHC: three-dimensional conformations of gallic acid (a), caffeic acid (c), and *p*-hydroxybenzoic acid (e); magnified three-dimensional views of the binding sites for gallic acid (b), caffeic acid (d), and *p*-hydroxybenzoic acid (f).

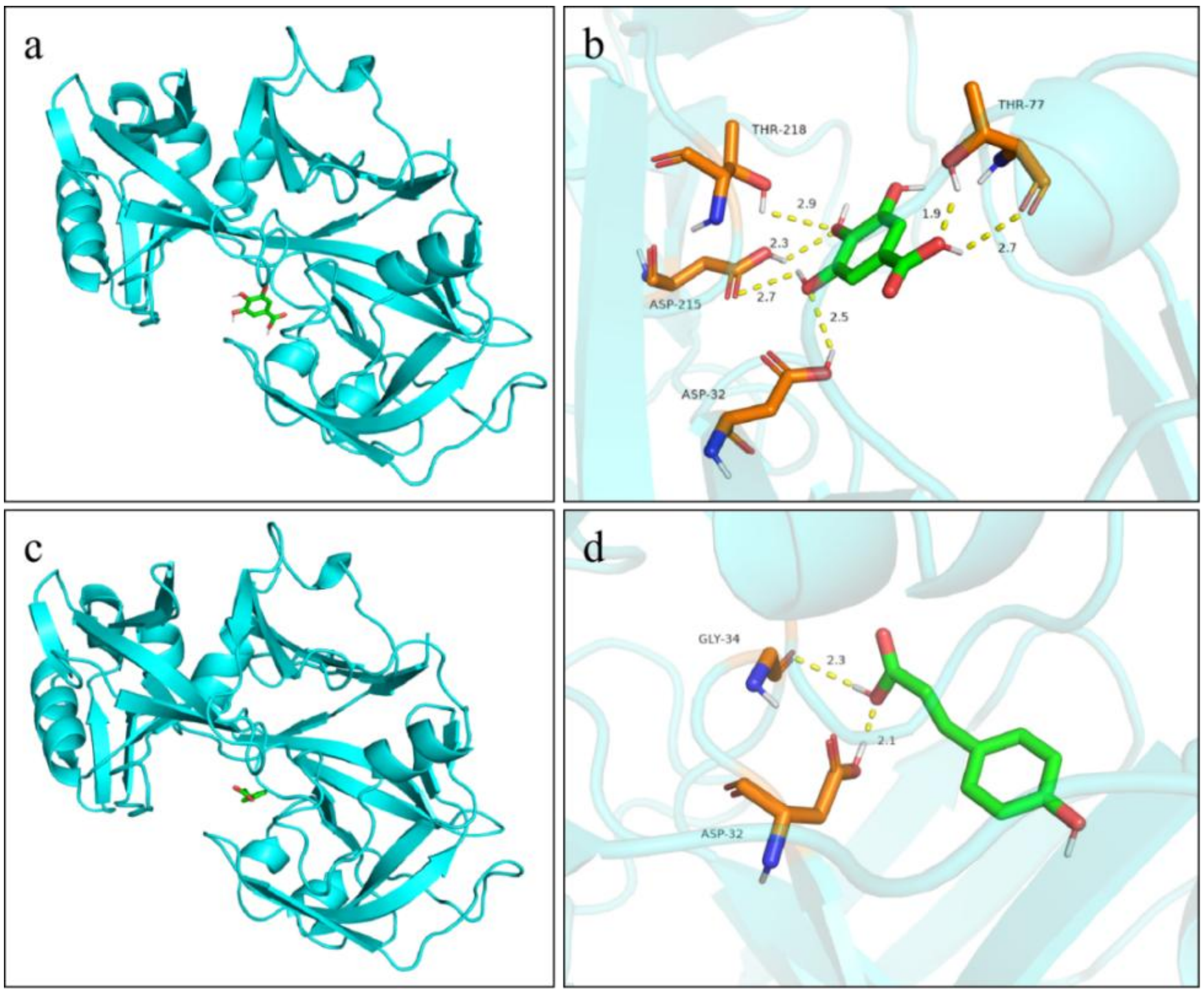

Fig.S6 Molecular docking results of two representative phenolics with pepsin: three-dimensional conformations of gallic acid (a) and *p*-coumaric acid (c); magnified three-dimensional views of the binding sites for gallic acid (b) and *p*-coumaric acid (d).

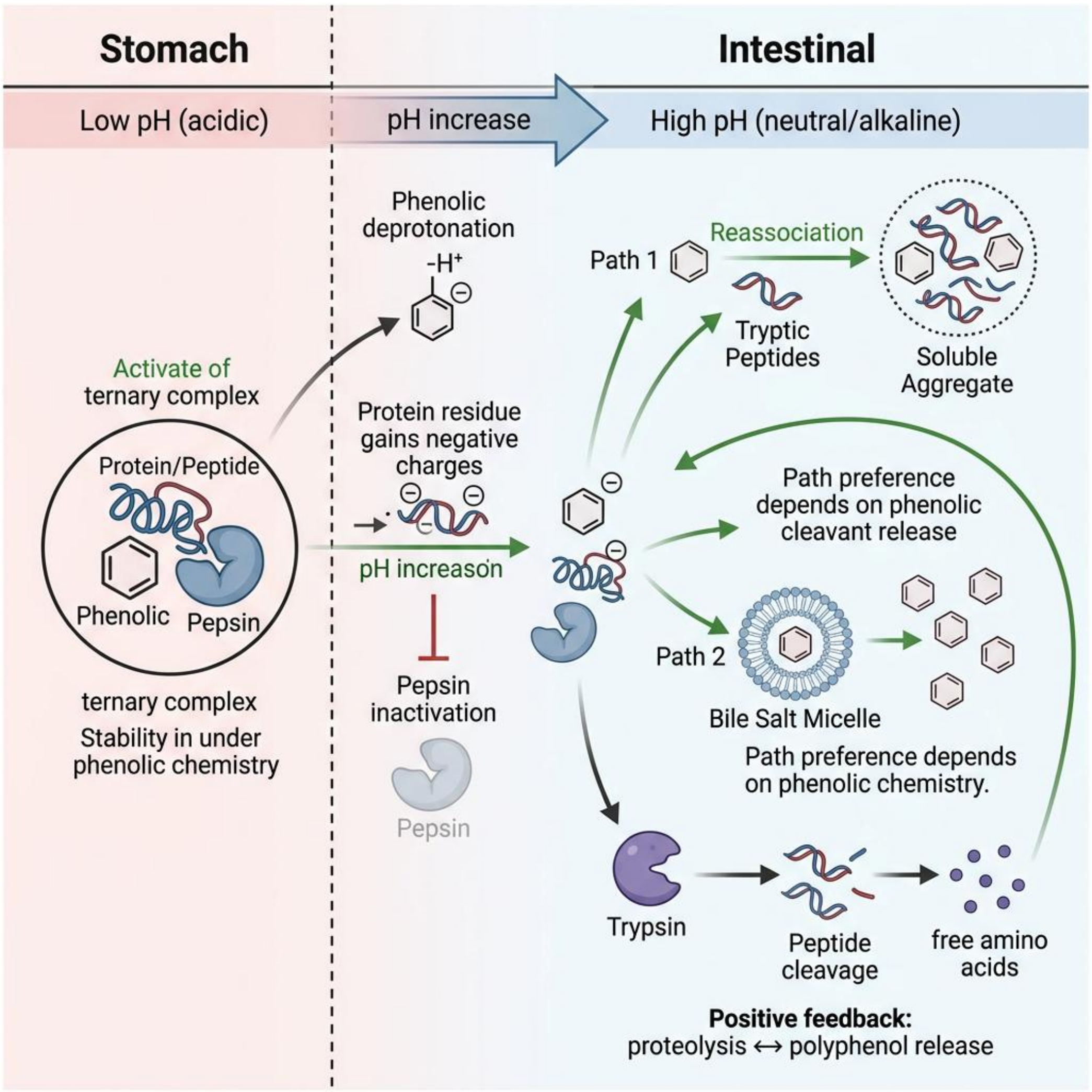

Fig.S7 Proposed model of ternary complex dissociation and phenolic redistribution in the beef–wine co-digestion matrix during the intestinal phase.
